# Human decisions about when to act originate within a basal forebrain–nigral circuit

**DOI:** 10.1073/pnas.1921211117

**Published:** 2020-05-08

**Authors:** Nima Khalighinejad, Luke Priestley, Saad Jbabdi, Matthew F. S. Rushworth

**Affiliations:** ^a^Wellcome Centre for Integrative Neuroimaging, Department of Experimental Psychology, University of Oxford, Oxford OX1 3SR, United Kingdom;; ^b^Wellcome Centre for Integrative Neuroimaging, Centre for Functional Magnetic Resonance Imaging of the Brain, University of Oxford, John Radcliffe Hospital, Oxford OX3 9DU, United Kingdom

**Keywords:** self-initiated action, basal forebrain, ultrahigh-field MRI, structural equation modeling, human

## Abstract

Decisions about when to act are critical for survival in humans as in animals, but how a desire is translated into the decision that an action is worth taking at any particular point in time is incompletely understood. Here we show that a simple model developed to explain when animals decide it is worth taking an action also explains a significant portion of the variance in timing observed when humans take voluntary actions. The model focuses on the current environment’s potential for reward, the timing of the individual’s own recent actions, and the outcomes of those actions. We show, by using ultrahigh-field MRI scanning, that in addition to anterior cingulate cortex within medial frontal cortex, a group of subcortical structures including striatum, substantia nigra, basal forebrain (BF), pedunculopontine nucleus (PPN), and habenula (HB) encode trial-by-trial variation in action time. Further analysis of the activity patterns found in each area together with psychophysiological interaction analysis and structural equation modeling suggested a model in which BF integrates contextual information that will influence the decision about when to act and communicates this information, in parallel with PPN and HB influences, to nigrostriatal circuits. It is then in the nigrostriatal circuit that action initiation per se begins.

## Significance

Decision-making studies often focus on brain mechanisms for selecting between goals and actions; however, another important, and often neglected, aspect of decision-making in humans concerns whether, at any given point in time, it is worth making any action at all. We showed that a considerable portion of the variance in when voluntary actions are emitted can be explained by a simple model that that takes into account key features of the current environment. By using ultrahigh-field MRI we identified a multilayered circuit in the human brain originating far beyond the medial frontal areas typically linked to human voluntary action starting in the basal forebrain and brain stem, converging in the dopaminergic midbrain, and only then projecting to striatum and cortex.

Flexible behavior involves not only choosing the right action but also initiating the chosen action at just the right moment. For example, a hunting animal must strike at just the right time, when it is close enough to reach its prey before it has a chance to escape but when it is still far enough away to avoid detection. The same is true in humans; an art collector may choose to bid for a specific item in an auction, but it is also important to place the bid at the right moment, when other bidders will not have a chance to outbid, but still leave enough time to place a bid. The ability to initiate self-timed actions is vital to animals’ survival, and the consequences of its disruption can be observed in neurological disorders such as Parkinson’s disease. It is well established that free voluntary choices in humans depend in some way on a brain region somewhere in the medial frontal cortex, but an explanation of why decisions to act emerge at particular points in time has been lacking (1–4).

We have recently shown in macaques that a circuit comprising anterior cingulate cortex (ACC) and basal forebrain (BF) integrates past and present contextual information that influences when an action is made (5). However, the interplay between BF and other brain circuits involved in the generation of self-initiated actions remains unclear. Basal ganglia, in particular, play a major role in different aspects of action selection and initiation (6, 7); activity in many striatal neurons increases prior to action onset (8, 9). Additionally, activity in substantia nigra (SN) pars compacta (SNc) dopamine neurons, and their terminals in the striatum, has been linked to self-paced action initiation (10, 11). Our previous finding that BF mediates the influence of past and present context on the emergence of a decision about when to act might therefore seem surprising, especially given that BF is the major source of cholinergic projection neurons to cortex. However, it has been suggested that acetylcholine may also play an independent and complementary role in initiation of self-timed actions (12). For example, the activity of the cholinergic neurons that project from the pedunculopontine nucleus (PPN) to the SNc dopamine neurons modulates locomotion (13). However, we still know comparatively little about the interplay between BF and other subcortical nuclei associated with dopamine and the striatum. The first major aim of the current study was to elucidate this relationship and to test whether BF integrates information about when an action should be made while activity in interconnected nuclei is linked to the action initiation per se.

The second major aim was to investigate these processes in the human brain. We used a behavioral paradigm to investigate in humans how contextual factors and internal state, shaped by present and past environment, are integrated to determine when to act. We used ultrahigh-field functional magnetic resonance imaging (7T fMRI) of cortical and subcortical structures to identify brain activity mediating decisions about when to act. Finally, we used psychophysiological interaction (PPI) analysis and structural equation modeling (SEM) to examine the interaction between these structures at a circuit level.

First, behavioral analyses demonstrated that in humans, contextual information influenced apparently voluntary decisions about when to act. As in macaques (5) a large proportion of variance in decisions about when to act could be explained by a quantitative model that deduced what we refer to as a deterministic component of time to act based on features of the environment relating to both current context and the recent past context. Second, ultrahigh-field functional imaging identified a group of subcortical structures whose activity was parametrically related to the factors that change the likelihood of action at a given point in time, rather than action initiation per se. Third, model-based fMRI analysis showed that as in macaques, blood-oxygen-level–dependent (BOLD) activity in BF could be explained by trial-to-trial variation in deterministic action time, which is the predicted action time given present and past contextual factors. In addition, however, the current behavioral paradigm also made it possible to identify other patterns of activity more directly linked to action initiation per se in other nuclei. Fourth, examination of the patterns of activity interaction across these nuclei, aided by PPI and SEM, allowed us to identify a multilayered circuit in the human brain originating far beyond the medial frontal areas typically linked to human voluntary action initiation, starting in the BF, habenula (HB), and PPN; converging in the dopaminergic SN; and only then projecting to striatum and cortex.

## Results

### Participants Used Contextual Factors to Decide When to Act.

**Fig. 1.**
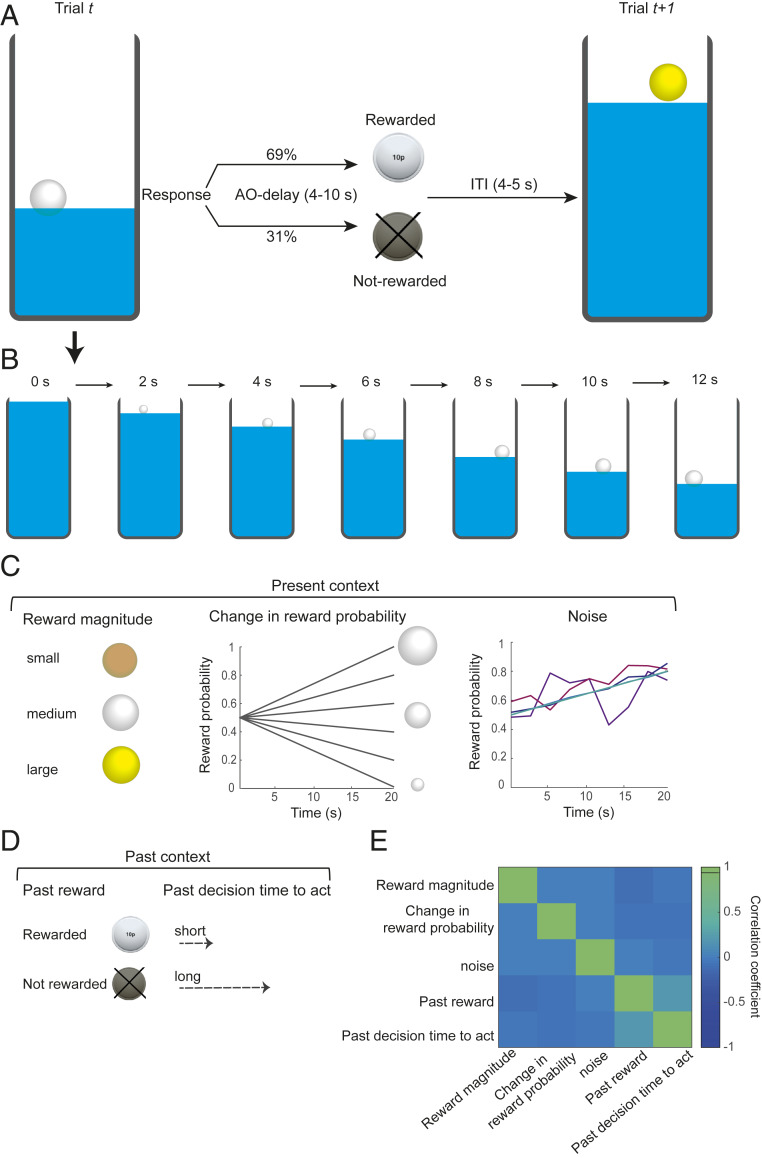
Experimental task. (*A*) At the beginning of each trial a vertical rectangle appeared on the center of the screen which we refer to as the water tank. The water (the blue filling of the rectangle) level started dropping as soon as the trial started. As the water level was dropping, bubbles (transparent circles) emerged from the water. Participants were told that bubbles might contain reward. The color and the size of bubbles represented potential reward magnitude and reward probability, respectively. Participants could choose a bubble by pressing on a response button at a time of their own choice. Once they responded, the stimulus disappeared, and participants waited for 4 to 10 s (action–outcome [AO] delay) before receiving the outcome. During the outcome phase, if rewarded, a gold, silver, or bronze coin was shown on the screen, representing 20, 10, or 5 p, respectively. If not rewarded, or in rare occasions that participants did not make any response, a dark coin appeared on the screen. (*B*) Timeline of one example trial. At the beginning of each trial a water tank filled with water was presented on the center of the screen. As the water level started dropping, bubbles emerged from the water, one at a time. Each bubble remained on the screen for 2 s before popping and a new bubble emerging. It took 20 s for the whole tank to drain (total number of bubbles in a tank = 9). In the example shown (trial *t*), silver bubbles (medium reward magnitude) emerge from the water. As the water level drops, bubbles are getting bigger and bigger, meaning that in this trial the change in reward probability slope is positive (first bubble, 50%; last bubble, 80%). In this example, the participant decides to respond after 12 s (with 69% chance of getting 10 p). (*C*) Contextual factors from the current and past trials were used to predict participants’ time to act. Present contextual factors consisted of reward magnitude (three levels) shown with different color of bubbles, rate of change in reward probability (six levels) shown with different size of bubbles, and white Gaussian noise added to the linearly changing bubble size (five levels) (in the figure, for clarity, noise levels are only added to one of the probability slopes). (*D*) Past contextual factors consisted of reward outcome (two levels) and *actTime* on the past trial (continuous variable). (*E*) Correlation matrix of present and past contextual factors. The contextual factors were varied from trial-to-trial, independently of one another, and in a pseudorandomized order.

We developed a task to investigate in humans how contextual factors and internal state, shaped by present and past environment, are integrated to determine when to act. Twenty participants were instructed to track stimuli on the screen (bubbles emerging from a draining water tank, one at a time, every 2 s) and to choose a bubble by making a response at a time of their choice (only one bubble could be picked per trial) (Fig. 1 *A* and *B*). Each bubble potentially contained a monetary reward. The magnitude and the probability of reward were represented by the color and the size of the bubble, respectively. The color and rate of change (slope) in bubble size changed from trial to trial but remained constant within a trial: gold, silver, and bronze bubbles contained large, medium, and small levels of reward; the bubbles got bigger and bigger (higher reward probability) or smaller and smaller (lower reward probability), as the water level was dropping, with different slopes. It took 20 s for the whole tank to drain. In addition, different levels of noise were added to the linearly changing bubble size: while in some trials it was very easy for participants to track the rate of change in size of the bubbles (reward probability), it was much harder in other trials. Together these factors comprised the present contextual factors (Fig. 1*C*) (*Methods*). Importantly, they were varied independently of one another and in a pseudorandomized order (Fig. 1*E*). Previous investigations have shown that the timing of the next action that a rat or macaque makes is related to the timing of recent previous actions (5, 14). Therefore, in addition to the present context, we also investigated whether the outcomes and action times of recent past trials influenced human participants’ action time on the current trial. These factors comprised the past contextual factors (Fig. 1*D*).

**Fig. 2.**
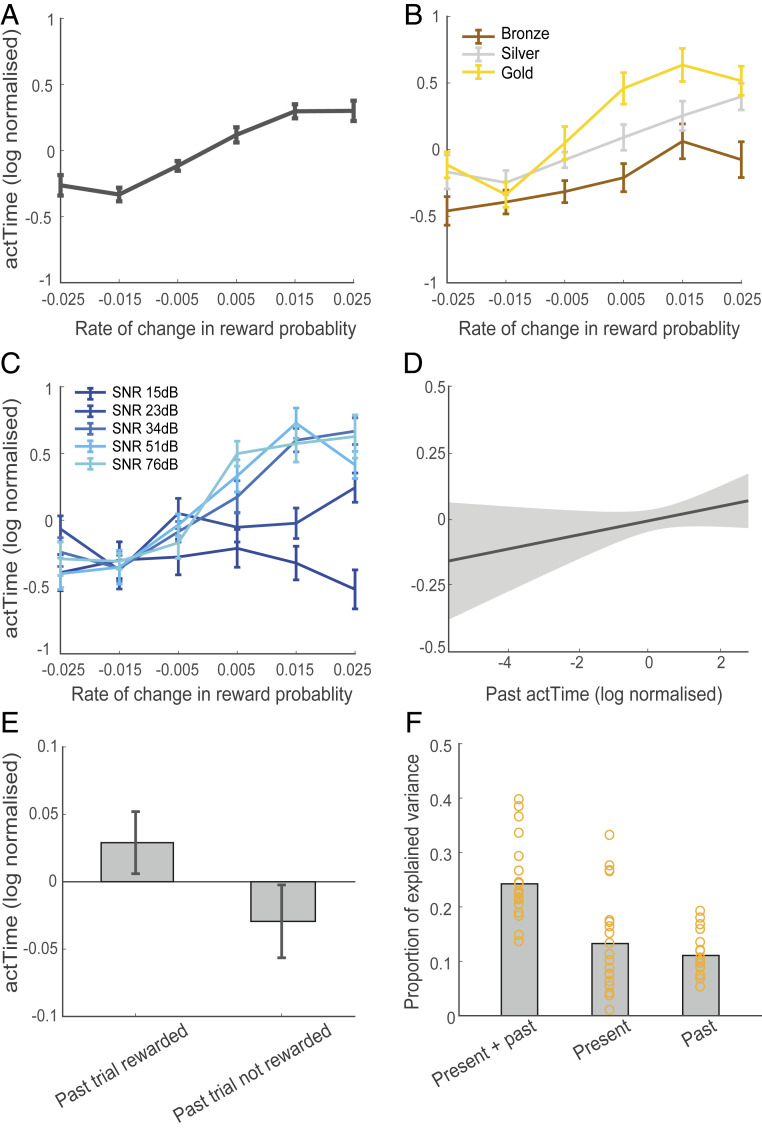
Present and past contextual factors influenced *actTime*. (*A*–*C*) Effect of present contextual factors on *actTime*. Participants waited before responding when bubbles were getting bigger (positive reward probability slope), but they acted quickly when bubbles were getting smaller (negative reward probability slope) (*A*) (see *SI Appendix*, Fig. S1, for individual participant data). The long *actTime* was more pronounced when participants were offered a large compared to a small reward (*B*) and when it was easy to track the rate of change in reward probability (low level of noise; SNR, signal-to-noise ratio) (*C*). On the *x* axis the rise is the difference between the reward probability in the last (0, 0.2, 0.4, 0.8, and 1) and first (0.5) bubble, and the run is the time it takes for the whole water tank to drain (20 s). (*D* and *E*) Effect of past contextual factors on *actTime*. Participants waited longer before responding when they had already delayed *actTime* on the past trial (*D*), and it was longer when they had received a reward compared to no reward on the past trial (*E*). Error bars show standard error of the mean across participants. In *D*, the shaded area is SE across observations. (*F*) Proportion of explained variance (PEV) in *actTime* explained by the Cox regression model. PEV is estimated separately from present and past, present, and past contextual factors. Each ring represents one participant. See also *SI Appendix*, Fig. S1.

We investigated whether these features of the present and past context influenced when humans decide to act. Time to act (*actTime*) was indexed as the time passed in seconds from the moment the water level started dropping until the participant made a response. On average, participants made a response after 9.75 ± 1.83 s (*SI Appendix*, Fig. S1*B*). A linear mixed-effect model (*SI Appendix*, *SI Methods*) showed a significant interaction between rate of change in reward probability and reward magnitude [β = 0.05 ± 0.02, *X*^*2*^ (1) = 6.85, *P* = 0.009] and noise [β = 0.15 ± 0.03, *X*^*2*^ (1) = 34.29, *P* < 0.0001]. This suggests that participants waited longer before responding when bubbles were getting bigger (positive reward probability slope), but they did so more when offered a large compared to a small reward and when it was easy to track the rate of change in size of the bubbles (low level of noise) (Fig. 2 *A*–*C*). Past contextual factors also influenced participants’ *actTime*. *actTime* on the current trial was longer when *actTime* had been longer on the past trial [β = 0.06 ± 0.03, *X*^*2*^ (1) = 4.63, *P* = 0.03; Fig. 2*D*], and it was longer when participants had received a reward compared to no reward on the past trial. However, this last effect did not reach significance [β = 0.09 ± 0.05, *X*^*2*^ (1) = 3.42, *P* = 0.06; Fig. 2*E*].

### Contextual Factors Explained a Large Proportion of Variance in Time to Act.

Having shown that contextual factors influence decision time to act, we next used a Cox proportional hazard model to estimate *actTime*, at each trial, from present and past contextual factors (5, 14). Specifically, we asked how much time (in seconds) would pass before a participant decided to respond, given present and past contextual factors. To make comparison across species possible we followed the same procedure as previously used to estimate *actTime* in macaques (5): First, we estimated Cox regression coefficients for reward magnitude, rate of change in reward probability, and noise on the current trial. In addition, we estimated Cox regression coefficients for actual reward outcome and *actTime* on past trials. Next, we used the Cox regression coefficients from present and past trial contextual factors (*t* and *t* − 1, where *t* is number of trial) to estimate the expected *actTime* at each trial. We refer to the prediction of the model as deterministic *actTime*_present__+__past_ _context_. In contrast to the *actTime* actually observed (observed *actTime*), the deterministic *actTime*_present__+__past_ _context_ is the time passed, from beginning of the trial, at which the participant is expected to make a response, given the influence that present and past contextual factors are known to have. Subsequently, we separately assessed the contribution of past and present context to deterministic *actTime*. We used the Cox regression coefficients relating to either the present trial (*t*) or the past trials (*t* – 1, similar to the original model) to derive two separate *actTime* estimates. These new estimates were termed deterministic *actTime*_present_ _context_ and deterministic *actTime*_past_ _context_.

We then asked what percentage of the trial-to-trial variability in observed *actTime* could be explained by present context, past context, or a combination of both contexts (*SI Appendix*, *SI Methods*). On average, present and past contextual factors together explained 24 ± 8% of *actTime* variance. Of this, 13 ± 9% and 11 ± 4% were explained by present and past contextual factors, respectively (Fig. 2*F*). This is lower than in a related paradigm in monkeys (36 ± 9%) (5) but still a large proportion of variance.

### A Subset of Subcortical Structures Encodes Decision Time to Act.

**Fig. 3.**
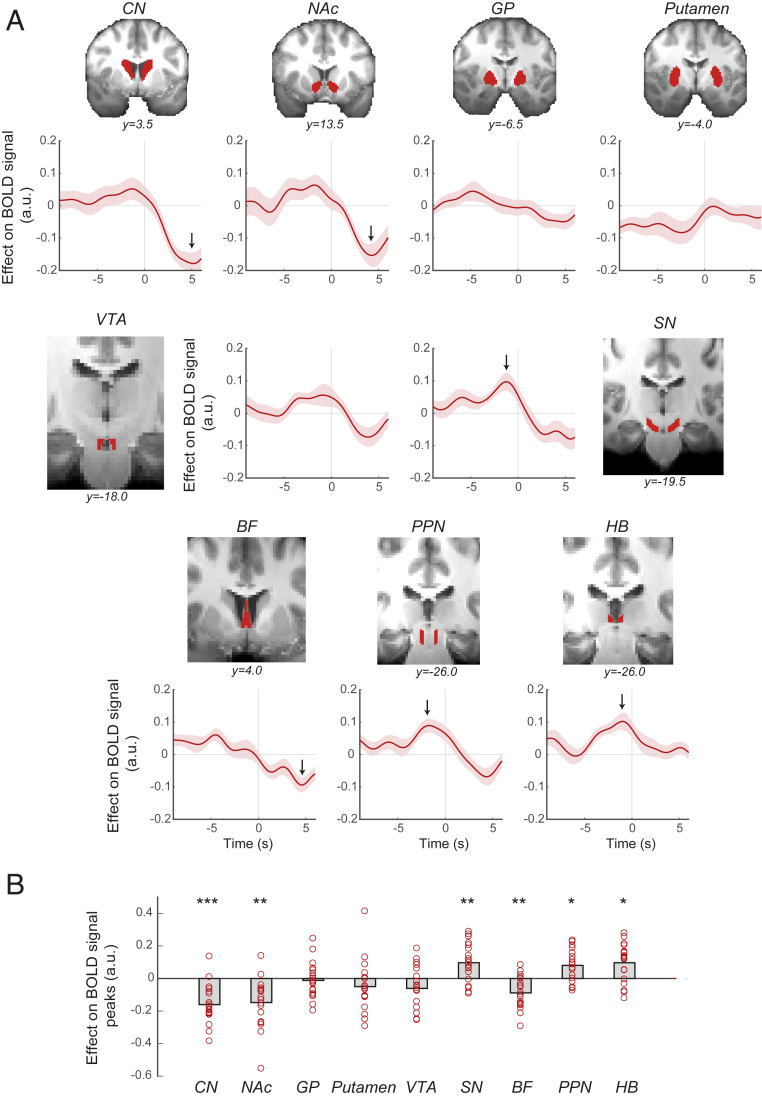
A subset of subcortical structures encodes decision time to act. (*A*) ROI time course analysis of the a priori selected subcortical structures, showing the relationship between BOLD and observed *actTime*. The panel next to each time course shows the corresponding anatomical ROI overlaid on averaged structural image of all subjects in standard space. The *y* axis is based on the FSL MNI152 standard brain in which *y* = 0 is the dorsal posterior corner of the anterior commissure (ac). Other commonly used atlases such as the Atlas of the Human Brain (43) put *y* = 0 at the center of ac. The lines and shadings show the mean and SE of the β weights across the participants, respectively. The arrows show the location of the peak effect. Time 0 is the response time. Note that the hemodynamic lag means that a BOLD signal change reflects neural activity ∼6 s earlier. (*B*) There was a significant relationship between BOLD activity and *actTime* in CN, NAc, SN, BF, PPN, and HB. However, given the delay in the hemodynamic response, it is clear that the activity in CN, NAc, and BF begins during a late decision phase just before the initiation of action; by contrast, SN, PPN, and HB encode *actTime* long before the initiation of action during an early decision phase. Each ring represents one participant. The gray columns illustrate the group mean. One-sample *t* tests with Holm–Bonferroni correction. *P < 0.05, **P < 0.01, ***P < 0.001. See also *SI Appendix*, Figs. S2–S4.

To identify potential subcortical structures that track the parametric variation in action time we examined activity in anatomical regions of interest (ROIs), which have been linked to action initiation (5, 7, 11, 13, 15, 16). The a priori selected ROIs included caudate nucleus (CN), putamen, nucleus accumbens (NAc), globus pallidus (GP), SN, ventral tegmental area (VTA), PPN, HB, and BF (note that because of the close adjacency of several small and diverse nuclei near the nucleus basalis, our BF region focuses on septal nuclei and part of the diagonal band of Broca) (Fig. 3*A*). To investigate whether subcortical structures track the parametric variation in either the empirically observed *actTime* recorded on each trial or the deterministic *actTime* at which actions were expected to be made on each trial given the known influence of the environmental context, we created anatomical masks for each ROI and each individual participant (*SI Appendix*, Fig. S2) and extracted the time course of the neural activation from each ROI, with respect to response onset (*Methods*).

The empirically observed *actTime* (*Methods*, general linear model 2.1 [GLM2.1]; *SI Appendix*, Fig. S3, illustrates time course of each contextual factor; *Methods*, GLM2.2; see *SI Appendix*, Fig. S4, for alternative analysis) explained BOLD activity in CN [one-sample *t* test; *t* (18) = −5.87, *P* = 0.0001, *d* = 1.35; all subsequent tests are corrected for multiple comparisons], NAc [*t* (18) = −4.28, *P* = 0.004, *d* = 0.98], SN [*t* (18) = 3.51, *P* = 0.009, *d* = 0.81], BF [*t* (18) = −3.99, *P* = 0.006, *d* = 0.92], PPN [*t* (18) = 3.65, *P* = 0.01, *d* = 0.84], and HB [*t* (18) = 3.64, *P* = 0.01, *d* = 0.83] (Fig. 3*B*). Although timing differences in BOLD signals must be interpreted with care, it is noteworthy that the peak effect of parametric variation in observed *actTime* on BOLD signal was much earlier in SN, PPN, and HB compared to CN, NAc, and BF. On average, the effect of observed *actTime* on BOLD activity was positive and peaked 1.04 s before the response in the former group. In the latter group, this effect was negative and peaked 4.66 s after the response (Fig. 3*A*). A 6-s difference in activity peaks is unlikely to be due solely to differences in BOLD hemodynamic response functions and instead suggests different roles for the areas in specifying when to act. Given the delay in the hemodynamic response, it is clear that the activity in CN, NAc, and BF begins during a late decision phase just before the initiation of action; by contrast, SN, PPN, and HB encode *actTime* long before the initiation of action (average *actTime* across all conditions and participants is 9.61 s) during an early decision phase when the factors determining action first become observable. In support of this, a two-way repeated-measures ANOVA showed a significant interaction effect of decision phase and ROI on group peaks [*F*(5,90) = 2.61, *P* = 0.03, *η*_*p*_^*2*^ = 0.13] (*SI Appendix*, *SI Methods*), suggesting that parametric variation in observed *actTime* was associated with a late, negative BOLD response in CN, NAc, and BF but an early, positive BOLD response in SN, PPN, and HB.

**Fig. 4.**
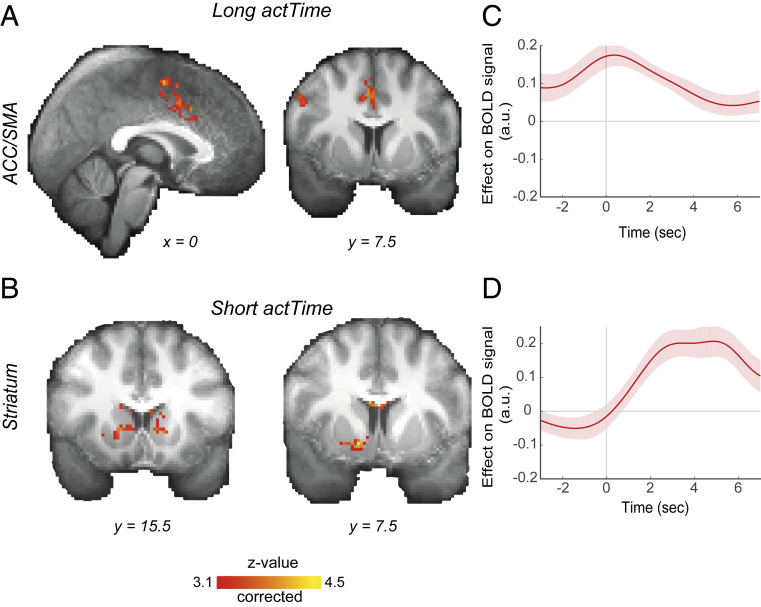
BOLD signal in ACC and striatum is correlated with time to act. Whole-brain analysis showing voxels where activity reflected parametric variation in the empirically observed *actTime*, on trials where rate of change in reward probability was (*A*) positive (*long actTime* was the correct strategy) and (*B*) negative (*short actTime* was the correct strategy). Whole-brain cluster-based correction, Z > 3.1. (See *SI Appendix*, Table S1 for the list of clusters.) (*C* and *D*) ROI time course analysis of the ACC/SMA (*C*) and striatum (*D*), showing the relationship between BOLD and *actTime*. Time 0 is the response time. Note that the hemodynamic lag means that a BOLD signal change reflects neural activity ∼6 s earlier. The lines and shadings show the mean and SE of the β weights across the participants, respectively. See also *SI Appendix*, Table S1.

Next, to identify cortical structures outside our anatomical ROIs that could also be involved in encoding of observed *actTime* we ran a whole-brain analysis. We used a GLM (*SI Appendix*, *SI Methods*, GLM1) to look for brain areas in which activity reflected parametric variation in the empirically observed *actTime*, separately on trials where rate of change in reward probability was positive (i.e., waiting longer before responding was associated with an increased chance of getting reward; long *actTime* contrast) and negative (i.e., responding quickly was associated with an increased chance of getting reward; short *actTime* contrast). For the long *actTime* contrast, the largest cluster was located in the ACC extending into supplementary motor area (SMA) (peak Z = 4.35, Montreal Neurological Institute [MNI] coordinate: *x* = 0, *y* = −4, *z* = 58; whole-brain cluster-based correction, Z > 3.1, *P* < 0.0001; Fig. 4*A* and *SI Appendix*, Table S1). For the short *actTime* contrast, the cluster was located at the striatum (peak Z = 4.42, MNI: *x* = 14, *y* = 8, *z* = −8; whole-brain cluster-based correction, Z > 3.1, *P* < 0.0001; Fig. 4*B* and *SI Appendix*, Table S1). Whole-brain analysis suggests that in addition to striatum, which was already part of our a priori selected ROIs, ACC and SMA are also involved in encoding of *actTime*. To illustrate the timing of encoding of observed *actTime* in ACC and striatum, we extracted the time course of the neural activation in a 14-mm^3^ sphere ROI centered on the activation peak with respect to response onset (Fig. 4 *C* and *D*). In accordance with our previous finding, the effect of *actTime* on BOLD signal in striatum peaked during the late decision phase. However, this effect peaked during the early decision phase in ACC.

### BF Communicates Decisions About When to Act to Nigrostriatal Pathway.

Time course analyses showed that BOLD response in a subset of our subcortical ROIs is correlated with parametric variation in empirically observed *actTime*. We next, however, asked 1) whether the same areas integrated contextual factors to compute the deterministic component of *actTime*, as estimated by the Cox regression model [specifically, based on our previous finding in macaques (5), we expected BF to be involved in encoding the deterministic *actTime*—the time at which the response is expected to be made given the known influence of the contextual factors], and 2) whether the same areas encoded action initiation per se, above and beyond the parametric variation in *actTime*.

**Fig. 5.**
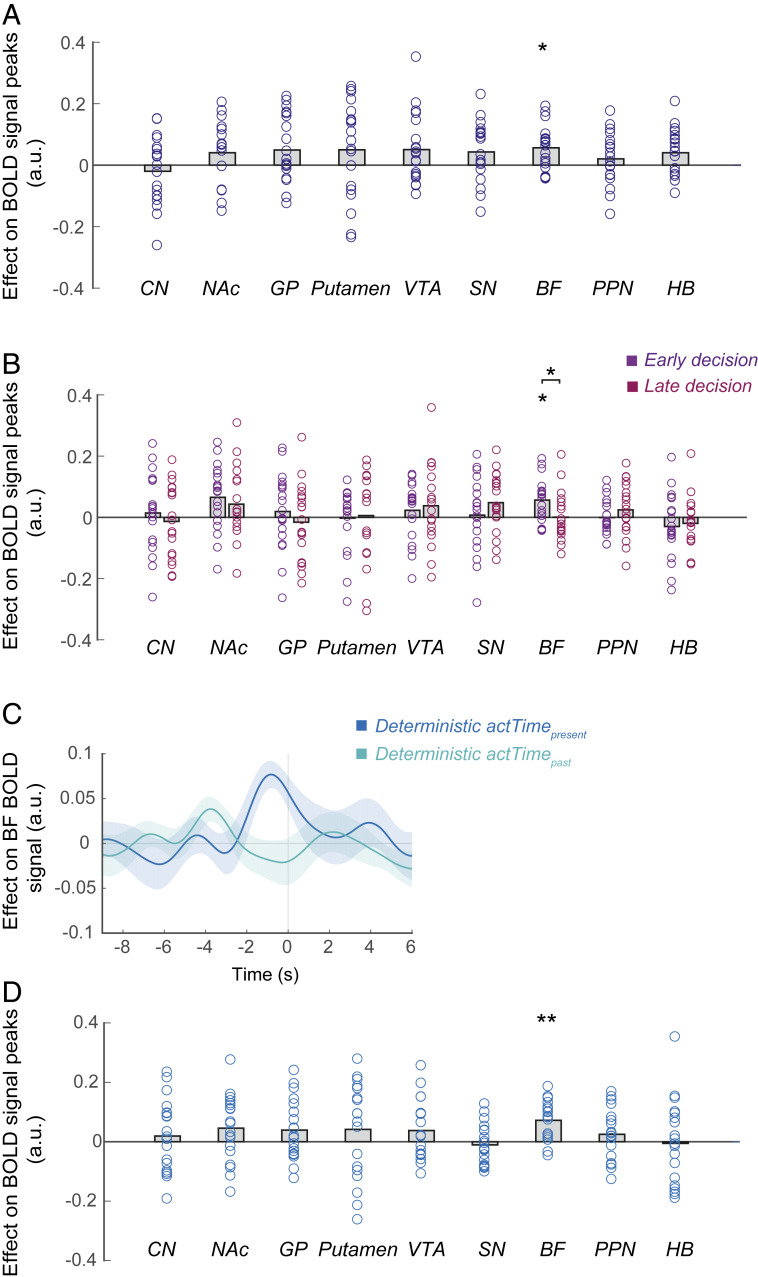
BF encodes the deterministic component of time to act. (*A*) Peak effect of deterministic *actTime* on ROI BOLD signal. Significance testing on time course data was performed by using a leave-one-out procedure on the group peak signal identified within the whole epoch. The effect of deterministic *actTime* on BF BOLD signal seems similar in size to effects in other areas. However, compared to other areas, deterministic *actTime* explained BOLD activity in BF in a uniform and consistent manner across participants and therefore in a significant way. (*B*) Peak effect of deterministic *actTime* on ROI BOLD signal identified separately within the early decision and late decision phases. (*C*) Time course analysis of the BF, showing the relationship between BOLD activity and deterministic *actTime* estimated separately from present (deterministic *actTime*_present_) and past (deterministic *actTime*_past_) context. Format is the same as in Fig. 3*A*. (*D*) Peak effect of deterministic *actTime*_*present*_ on ROI BOLD signal, identified within the whole epoch. Each ring represents one participant. The gray columns illustrate the group mean. Paired-samples *t* test and one-sample *t* tests with Holm–Bonferroni correction. *P < 0.05, **P < 0.01. See also *SI Appendix*, Figs. S4–S6.

To answer the first question, we added deterministic *actTime* to the time series GLM as the variable of interest and the observed *actTime* as covariate (*Methods*, GLM2.3). We found that deterministic *actTime* explained BOLD activity in BF [*t* (18) = 3.55, *P* = 0.02, *d* = 0.81; corrected for multiple comparisons]. This was not the case for other ROIs (Fig. 5*A* and *SI Appendix*, Fig. S5). This suggests that, as in macaques (5), BF activity in humans is involved in integrating present and past contextual information to construct the deterministic component of *actTime*. Interestingly, the effect of deterministic *actTime* on BF BOLD signal peaked during the early decision phase and was much earlier compared to the effect of observed *actTime* on BF BOLD (compare Figs. 3 and 5), and the effect was stronger during early compared to the late decision phase [paired-samples *t* test; *t* (18) = 2.33, *P* = 0.03, *d* = 0.53] (Fig. 5*B*). This suggests, after considering the BOLD hemodynamic lag, that deterministic *actTime* is encoded long before action initiation when the factors determining it become observable. Next, we asked whether present and past contextual factors contribute equally to encoding of deterministic *actTime* in BF. Deterministic *actTime*_present_ _context_ and deterministic *actTime*_past_ _context_ were used in a time series GLM (*Methods*, GLM2.4), with the observed *actTime* as covariate. BOLD activity in BF was related with deterministic *actTime*_present_ _context_ [*t* (18) = 4.87, *P* = 0.001, *d* = 1.12; corrected for multiple comparisons; *SI Appendix*, Fig. S6]. This was not true for *actTime*_past_ _context_ [*t* (18) = 2.22, *P* = 0.32; corrected for multiple comparisons*;* *SI Appendix*, Fig. S7]. This suggests that BF mostly employed present contextual factors to construct the deterministic component of *actTime* [paired-samples *t* test; *t* (18) = 2.00, *P* = 0.06, *d* = 0.46] (Fig. 5 *C* and *D*).

**Fig. 6.**
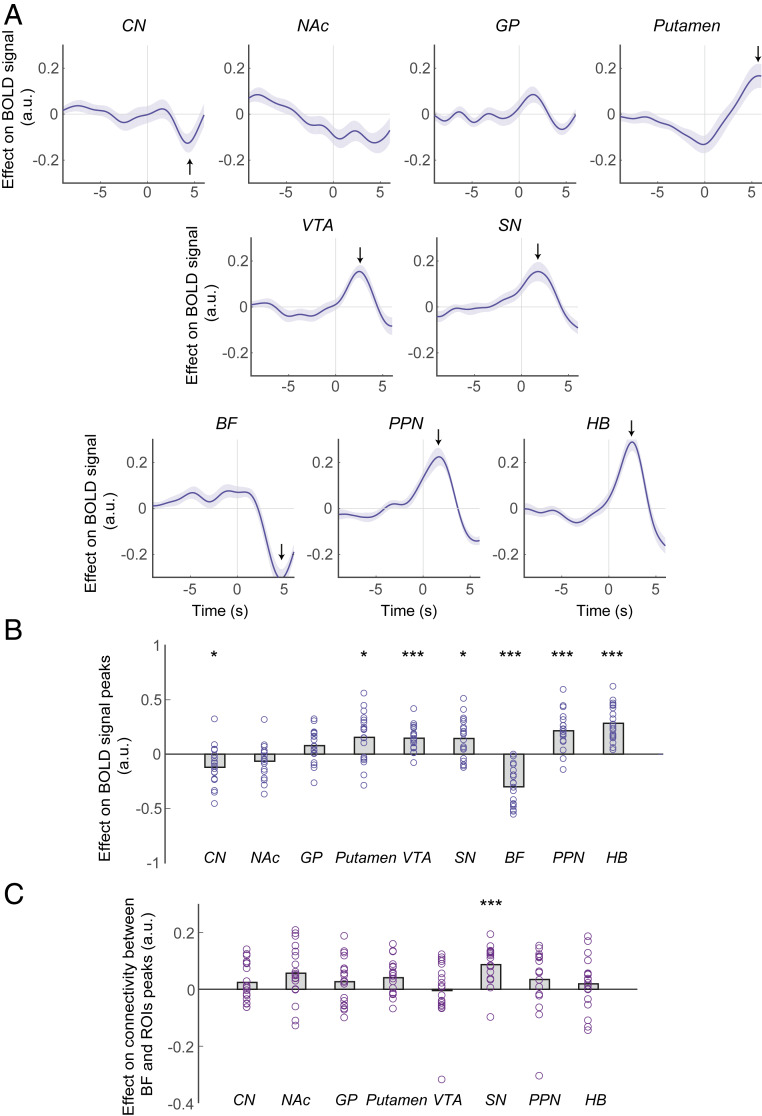
Dopaminergic midbrain, PPN, and HB encode action initiation per se. (*A*) ROI time course analysis of the ROIs, showing the relationship between BOLD activity and unmodulated action initiation. Format is the same as in Fig. 3*A*. The lines and shadings show the mean and SE of the β weights across the participants, respectively. Time 0 is the response time. Note that the hemodynamic lag means that a BOLD signal change reflects neural activity ∼6 s earlier. (*B*) Significance testing on time course data was performed by using a leave-one-out procedure on the group peak signal. (*C*) PPI analysis between BOLD signal in BF and other ROIs, with deterministic *actTime* as the psychological factor. Trial by trial variation in the activity in BF was significantly related with trial by trial variation in the activity in SN as a function of deterministic *actTime*. Significance testing on PPI data was performed by using a leave-one-out procedure on the group peak signal. Each ring represents one participant. The gray columns illustrate the group mean. One-sample *t* tests with Holm-Bonferroni correction. *P < 0.05, ***P < 0.001.

To answer the second question—whether the same areas encoded action initiation per se, above and beyond the parametric variation in *actTime*—we regressed an unmodulated regressor indexing action initiation against the ROIs’ extracted time series (*Methods*, constant regressor in GLM2.3) and added the deterministic and the observed *actTime* as covariates. Dopaminergic midbrain [one-sample *t* test corrected for multiple comparisons; SN, *t* (18) = 3.38, *P* = 0.02, *d* = 0.78; VTA, *t* (18) = 5.44, *P* = 0.0003, *d* = 1.25], PPN [*t* (18) = 5.47, *P* = 0.0003, *d* = 1.25], and HB [*t* (18) = 7.56, *P* < 0.0001, *d* = 1.73] showed a positive peak ∼2 s after initiation of action (Fig. 6 *A* and *B*), therefore, once the hemodynamic lag is taken into consideration, indicating activity prior to movement onset. This effect on BOLD response is constant and does not vary from trial to trial; it thus demonstrates the encoding of action initiation per se rather than parametric variation in observed or deterministic *actTime*. Interestingly, the BOLD response peaked at the same time in all four areas and showed a gradual ramp-up starting about 3 s before initiation of action, suggesting activity was present during the early decision phase. We also observed an effect in CN, putamen, and BF, but the peak occurred much later at about 6 s after the response onset, suggesting activity was present during the late decision phase.

So far, we have shown that BOLD response in CN, NAc, SN, BF, PPN, and HB is correlated with observed *actTime.* Among these areas, however, BF encoded the deterministic component of *actTime* while SN, PPN, and HB encoded the action initiation per se, during the early decision phase. We then asked whether BF is functionally connected with SN, PPN, or HB as a function of deterministic *actTime.* We performed a PPI analysis (17) (*Methods*, GLM2.5) and found that the functional connectivity between BF and SN is moderated by deterministic *actTime* [one-sample *t* test corrected for multiple comparisons; *t* (18) = 5.76, *P* = 0.0001, *d* = 1.32]. This was not true for the functional connectivity between SN and the other ROIs (Fig. 6*C*). Stronger activity in BF was associated with stronger activity in SN as a function of deterministic *actTime*, compared to functional connectivity between BF and PPN [paired-samples *t* test; *t* (18) = 2.55, *P* = 0.02, *d* = 0.58] or between BF and HB [*t* (18) = 2.32, *P* = 0.03, *d* = 0.53]. This is consistent with the BF communicating decisions about when to act to the nigrostriatal pathway. It is then within the nigrostriatal circuit or one of the interconnecting areas such as PPN or HB that action initiation per se begins.

### Decisions About When to Act Are Constructed Within a Cortico-Subcortical Circuit.

There was a discernible pattern in the type and timing of BOLD signals in subcortical ROIs; the peak effects of deterministic *actTime* in BF and observed *actTime* in the SN, PPN, and HB tended to arise in the first few seconds of the trial (early decision phase) and were followed by peak effects in striatum just before action initiation (late decision phase). Although inferences about the timing of neuronal activity from BOLD response should be treated with caution, the pattern of activity in our ROIs, and previous work on the direct or indirect pathways between them (7, 15), is suggestive of a cortico-BF–midbrain–striatal circuit for decisions about when to act. Therefore, we tested whether interrelationships in the time series of BOLD signals from our ROIs indicated a circuit within this structure. This was done by fitting an SEM to time series data from ROIs that were involved in encoding of *actTime*. SEMs define the strength of connections between brain areas in question rather than the degree of activity relating to individual behavioral variables (*SI Appendix*, *SI Methods*). As a result, whether or not an individual ROI encodes *actTime* is orthogonal to the question of its functional interactions with other ROIs.

**Fig. 7.**
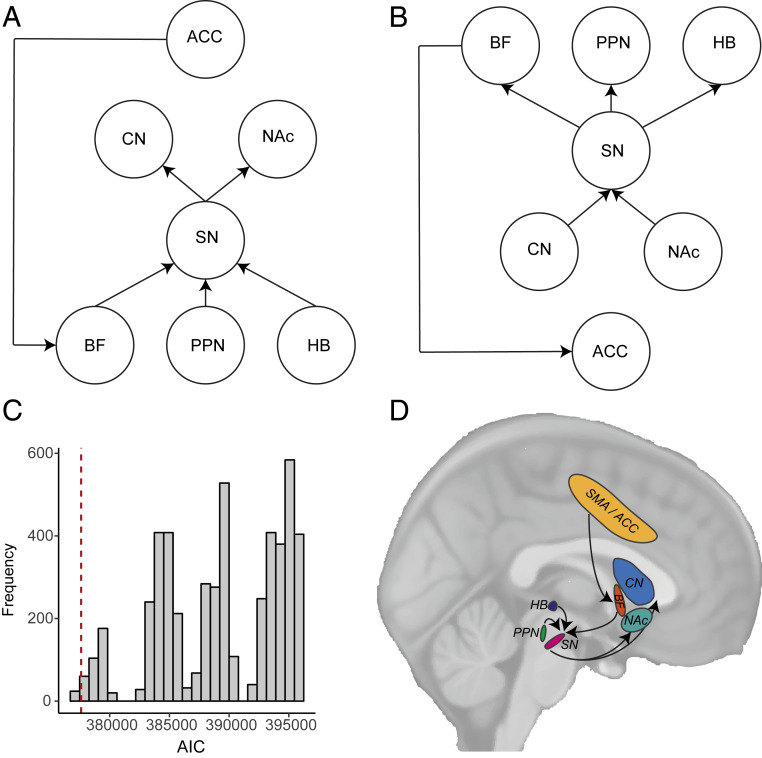
Decisions about when to act are constructed within a cortico-subcortical circuit. (*A*) The hypothesized model in which activity in CN and NAc is influenced by SN; activity in SN is influenced by BF, PPN, and HB; and ACC influences BF (for estimates of path coefficients, see *SI Appendix*, Table S2). (*B*) Alternative model. The hypothesis-driven model fits the data better than an alternative model in which the directions of paths were reversed. (*C*) Randomly permuting the position of each ROI in the hypothesis-driven model produced a distribution of AICs. The AIC of the hypothesis-driven circuit (dashed red line) was positioned in the 0.6th percentile of this distribution. (*D*) A schematic of a cortico-subcortical circuit for decisions about when to act. See also *SI Appendix*, Fig. S8.

First, based on the type and timing of BOLD signals in subcortical ROIs, we assumed a model in which activity in CN and NAc is influenced by SN; anatomical connections projecting from SN to CN and NAc and the influence SN exerts on CN and NAc are well known (18). We assumed that activity in SN is influenced by BF in line with the results of our PPI analysis (Fig. 6*C*). We also included influences form PPN and HB to SN in line with previously reported monosynaptic projections from PPN and HB to SN (15). We also included in the model an influence from ACC to BF; BF receives a monosynaptic input from ACC, and they are known to act in concert to determine *actTime* in monkeys (5, 19, 20) (Fig. 7*A*; see also *SI Appendix*, Fig. S8). Next, we estimated the path coefficients to find out whether the data we had observed would fit the model. As predicted, all specified path coefficients in the hypothesized model were significantly different from zero (*SI Appendix*, Table S2) and provided a good description of the data according to at least two of the standard fit indices for structural equation models (standardized root mean square residual = 0.066; goodness-of-fit index = 0.961; root mean square error of approximation = 0.087; *SI Appendix*, *SI Methods*).

Having established that our proposed model fits the observed data well, we compared our model with alternative models and performed a series of control analyses to investigate which connections are influencing the network most. First, given that all brain areas have manifold connections, null models presuming no connections between ROIs are unsuitable points of comparison for connectivity analysis (21). Therefore, we compared the hypothesis-driven model to an alternative model in which the direction of connections was reversed (Fig. 7*B*). Note that the alternative model therefore has an identical number of degrees of freedom. The hypothesis-driven model (Akaike information criterion [AIC] = 377,600.5) provided a better description of the data than the alternative model (AIC = 486,306.6).

Second, to rule out the possibility that the significant path coefficients in the hypothesis-driven model are due to factors unrelated to *actTime*, we compared the hypothesized model against an alternative in which parametric variation in BOLD signal due to observed *actTime* was regressed out. This was done by convolving the main effect of responding and the parametric *actTime* (time-locked to response) with the canonical HRF and feeding these variables as inputs to all ROIs—the idea being that any remaining variance after these inputs reflects variance over and above the effects of observed *actTime*. The hypothesis-driven model (AIC = 377,600.5) provided a better description of the data than the alternative in which *actTime*-relevant variance was removed (AIC = 673,504.8). This supports the idea that the paths shown in the hypothesized model are, indeed, interactions that occur as a function of action timing.

Third, we performed a complementary test by comparing the hypothesis-driven model to other models of equivalent complexity by randomly permuting the position of each ROI in the circuit and obtaining the AIC of each variation. Again, this ensures that the alternative models have identical numbers of degrees of freedom. This yielded a total of 5,040 models with a median AIC of 389,479.2 (interquartile range = 9,209.4; range = [377,269.6 to 396,045.7]). The AIC of the hypothesis-driven model was 377,600.5, which positioned it in the 0.6th percentile of the distribution (Fig. 7*C*).

## Discussion

Previous work in humans has identified medial frontal brain areas associated with self-generated, self-timed, or voluntary actions (1, 4). Animal studies on the other hand have emphasized the role of basal ganglia circuits (6, 7). On the basis of the current results, we propose a circuit comprising structures in medial frontal cortex, basal ganglia, brainstem, and BF, working in concert to encode decisions about when to act and then actually initiating the action (Fig. 7*D*).

Participants performed a behavioral task, while inside an ultrahigh-field MRI scanner. They integrated contextual factors, shaped by the present and past environment, that influenced when they would act. We then used functional imaging to look for brain activity parametrically related to the factors that determine when might be the right time to make the action. We found that activity in ACC, CN, NAc, SN, PPN, HB, and BF encodes parametric variation in *actTime.* Self-initiated actions have previously been associated with medial frontal areas such as ACC and SMA (22–24) and basal ganglia such as CN, NAc, and SN (7, 11). However, the possibility that PPN, HB, and BF have roles in self-initiated action has received less attention.

BOLD activity in BF was correlated with deterministic *actTime* on each trial, providing the first piece of evidence that it was important for action timing. The activity change peaked ∼1 s before the actual response was made (Fig. 5*A*). Once the hemodynamic lag is taken into account, it is clear that BF activity occurs long before actual initiation of action and instead occurs at the point in time when visual cues indicating the contextual features that would influence action time were first presented. This early timing and the fact that—unlike any of the other brain areas investigated—its activity could be explained by the predicted *actTime* given the influence of present and past contextual factors (deterministic *actTime*) suggest an important role for the BF in mediating the influence of past and present context on decisions about when to act. This is consistent with previous studies in macaques (5).

The SN appears to be an important next stage in the circuit. Unlike BF, we did not find any evidence that SN activity reflects the contextual factors influencing when an action was likely to be made; its activity was not significantly related to deterministic *actTime*. However, its activity encoded action initiation per se and occurred early in trials prior to movement onset. Importantly, PPI analysis showed that functional connectivity between BF and SN was driven by deterministic *actTime*. It had been suggested that BF represents combinations of task-relevant contextual variables (25–28) and encodes decision time to act (5). However, it was not clear how these representations come to influence action time. Here we observed increased connectivity between BF and SN as a function of deterministic *actTime*, consistent with the idea that BF influences the nigrostriatal pathway implicated in self-initiated actions.

BF is not the only region to influence SN and the nigrostriatal pathway. HB and PPN also exhibited activity correlated with the empirically observed *actTime*, and similar to SN, they also encoded action initiation early in the trial. However, again unlike BF, we did not find any relation between HB and PPN activity and deterministic *actTime* suggesting they may exert distinct influences on SN. Hikosaka and colleagues describe similar patterns of activity in single neurons of the macaque HB and PPN, both of which encode motivational salience signals in response to newly encountered situations (29, 30). In the case of HB, these signals covary with the speed of accompanying saccades (29), suggesting that early onset activity—like the a*ctTime* signals observed here—might reflect updates to the participants’ estimates of key environmental features at the beginning of each trial (16, 31, 32). These updates might then be translated into adaptive control of downstream SN neurons at or around the time of action initiation. The PPN appears important for orienting behavior to the most rewarding course of action because lesions reduce the frequency of win–stay but not lose–shift patterns in rodent behavior (16, 33) (note the relationship between past reward outcome and PPN activity in *SI Appendix*, Fig. S3*D*). The HB, in contrast, may be linked to avoidance of negative outcomes and control of impulsive behaviors (15) or when a loss or an aversive event is predicted (note the relationship between expected reward on the current trial, *actTime*, and HB activity in *SI Appendix*, Fig. S3 *A* and *G*). Lesions of PPN or HB both induce changes in motor behavior, albeit different in nature, that are consistent with roles in action initiation (34–37).

Dopaminergic pathways have usually been associated with self-initiated action (11, 38). It is therefore noteworthy that two of the ROIs involved in action timing are distinguished by their cholinergic nature: the PPN, as a principal source of acetylcholine to the basal ganglia (39), and the BF, which is implicated in cholinergic neuromodulation of the cortex (40, 41). However, there is evidence for acetylcholine’s involvement in self-initiated action: The bradykinetic deficits of Parkinsonism are accompanied by degeneration of cholinergic neurons in the PPN (42), and the same population is important in PPN’s interactions with the nigrostriatal pathway (13).

Even though ultrahigh-field fMRI enabled us to extract BOLD signals from small structures in the BF, midbrain, and brainstem that would not have been possible with conventional methods, there are still limits to its spatial resolution and thus our ability to distinguish different neural populations. SN, for example, consists of the pars compacta and pars reticulata subdivisions that contribute to functionally distinct basal ganglia pathways (18), which we did not discriminate. BF includes various structures and nuclei such as the medial septal nucleus, diagonal nucleus, and nucleus basalis. However, because of the close adjacency of several small and diverse nuclei near the nucleus basalis, our BF region focuses on medial septal and diagonal nuclei (but see *SI Appendix*, Fig. S8). While neurophysiological recording is necessary for making such comparisons, fMRI can provide a simultaneous overview of activity across a distributed circuit.

Given the direct and indirect paths that are known to exist within basal ganglia circuits and the findings from our time course analyses, we proposed a circuit in which striatum is influenced by SN and SN is influenced by BF, PPN, and HB. We used structural equation modeling to verify the plausibility of such a circuit. We found that our hypothesized model fits the data well and performs better than alternative models (Fig. 7 and *SI Appendix*, Table S2). We do not, however, claim to be proposing a comprehensive model containing all functional connections between subcortical structures. There are, of course, other anatomically reasonable connections between structures of our proposed circuit, such as direct influences of the ACC onto SN and HB. However, we believe that our proposed model is the simplest anatomically plausible model that can explain our data well. We suggest that BF integrates past and present contextual information that will influence the decision about when an action should be made and communicates this information to nigrostriatal circuit (Fig. 6). It is then in the nigrostriatal circuit or one of the interconnecting areas such as PPN or HB that action initiation per se begins. On the other hand, medial frontal areas such as ACC might provide BF with contextual information it needs to guide decision time (Fig. 4) (5, 20). We found an influence from ACC to BF that may correspond with such a possibility during circuit-level analysis (Fig. 7).

## Methods

### Subjects.

Twenty participants (15 females), aged 19 to 34 y, completed the study. All participants were paid £10 per h for participating in the study and additional £3 to 7 for performance-dependent reward collected during the task. Each participant provided written informed consent at the beginning of the testing session. Ethical approval was given by the Oxford University Central University Research Ethics Committee (Ref-Number MSD-IDREC-R55856/RE001). One person was excluded from all neural analyses due to excessive head motion (absolute mean displacement > 2 mm). Behavioral data from all participants were included in analyses.

### ROI Time Course Analyses.

Anatomical ROIs were created in four stages for subcortical structures: 1) Anatomical masks were designed for each ROI in the MNI standard space using the Harvard–Oxford Subcortical Structural Atlas and Atlas of the Human Brain (43). 2) Masks were transformed from the standard space to each participant’s structural space by applying a standard-to-structural warp that was then thresholded, and binarized. 3) To make sure that the masks still match the ROIs’ boundaries after unwarping, they were manually edited within each participant’s structural space using FSLeyes. 4) Masks were transformed from the individual structural to functional space by applying a structural-to-functional warp, thresholded, binarized, and dilated by 1 voxel. Functional ROI (ACC and striatum) were defined as spheres of 1.5 mm radius, centered at the peak of the activation of a contrast. To avoid any circularity in analyses, functional ROIs were not used in time series analysis of *actTime* contrast.

For time series analyses, the filtered time series of each voxel within each ROI was averaged, normalized, and up-sampled. The up-sampled data were then epoched in 15-s windows, starting from 9 s before to 6 s after the response time. Time series GLMs were then fit at each time step of the epoched data, using ordinary least squares. We ran the following GLMs:

$$\text{GLM}2\text{.}1BOLD=\beta_{1}observed\_actTime+\beta_{2}totaltime+\beta_{3}constant,$$

where BOLD is an *i* × *t* (*i* trial, *t* time samples) matrix containing the times series data for a given ROI. *observed_actTime* is the time passed in seconds (log normalized) from beginning of the trial to the moment participants made a response. *totaltime* is a confounding regressor and accounts for the time passed since the beginning of the scanning session. *constant* is an unmodulated constant regressor.

$$\text{GLM}2\text{.}2BOLD=\beta_{1}reward_{t}+\beta_{2}probChange_{t}+\beta_{3}noise_{t}+\beta_{4}rewardOutcome_{t-1}+\beta_{5}actTime_{t-1}+\beta_{6}rewardOutcome_{t}+\beta_{7}totalTime_{t}+\beta_{8}constant,$$

where *reward*_*t*_, *probChange*_*t*_, and *noise*_*t*_ are contextual factors on the current trial; *rewardOutcome*_*t-1*_ and *actTime*_*t-1*_ are contextual factors on the past trial; and *rewardOutcome*_*t*_ is the reward outcome on the current trial.

$$\text{GLM}2\text{.}3BOLD=\beta_{1}deterministic\_actTime_{present+past}+\beta_{2}observed\_actTime+\beta_{3}totaltime+\beta_{4}constant,$$

where *deterministic_actTime*_*present+past*_ is the predicted *actTime* from the Cox regression model relating to both present and past contextual factors.

$$\text{GLM}2\text{.}4BOLD=\beta_{1}deterministic\_actTime_{present}+\beta_{2}deterministic\_actTime_{past}+\beta_{3}observed\_actTime+\beta_{4}totaltime+\beta_{5}constant,$$

where *deterministic_actTime*_*present*_ and *deterministic_actTime*_*past*_ are the predicted *actTime* from the Cox regression model relating to present and past contextual factors, respectively.

$$\text{GLM}2\text{.}5BOLD_{ROI}=\beta_{1}BOLD_{seed}+\beta_{2}deterministic\_actTime_{present+past}+\beta_{3}PPI+\beta_{4}observed\_actTime+\beta_{5}totaltime+\beta_{6}constant,$$

where $BOLD_{ROI}$ is BOLD activity at ROIs, $BOLD_{seed}$ is BOLD activity at BF, and *PPI* is the interaction between $BOLD_{seed}$ and *deterministic_actTime*_*present+past*_.

### Leave-One-Out Analysis on Time Series Group Peak Signal.

Significance testing on time course data was performed by using a leave-one-out procedure on the group peak signal to avoid potential temporal selection biases. For every participant, we estimated the peak signal time by identifying the peak in the time course of the mean beta weights of the relevant regressor in all other participants. When we did this, we identified the peak (positive or negative) of the regressor of interest within the full width of the epoched time course: from 9 s before to 6 s after the response. Next, we took the beta weight of the remaining participant at the time of the group peak. We repeated this for all participants. Therefore, the resulting 19 peak beta weights were selected independently from the time course of each single participant. We assessed significance using *t* tests on the resulting peak beta weights. To control for familywise error rate the significance level was adjusted for the number of ROIs, using the Holm–Bonferroni method (44). The effect of observed *actTime* on BOLD activity peaked 4.66 s after the response in one group of ROIs and 1.04 s before the response in another group. To further assess the significance of this timing difference we identified the (positive or negative) group peak within an early decision phase defined as a 2-s window before response and within a late decision phase defined as a 2-s window staring 4 s after the response. A leave-one-out procedure was used to identify group peak signals in both early and late decision phase.

### Materials and Data Availability.

Data files and materials used in the main analyses presented here have been archived and uploaded to the Data DRYAD and are freely available at https://doi.org/10.5061/dryad.prr4xgxhv (45).

## Supplementary Material

Supplementary File

## References

[1] Haggard P., Human volition: Towards a neuroscience of will. Nat. Rev. Neurosci. 9, 934–946 (2008). 19020512 10.1038/nrn2497

[2] Khalighinejad N., Schurger A., Desantis A., Zmigrod L., Haggard P., Precursor processes of human self-initiated action. Neuroimage 165, 35–47 (2018). 28966084 10.1016/j.neuroimage.2017.09.057 PMC5737384

[3] Lau H. C., Rogers R. D., Haggard P., Passingham R. E., Attention to intention. Science 303, 1208–1210 (2004). 14976320 10.1126/science.1090973

[4] Passingham R., The Frontal Lobes and Voluntary Action, (Oxford University Press, 1995).

[5] Khalighinejad N.., A basal forebrain-cingulate circuit in macaques decides it is time to act. Neuron 150, 370–384.e8 (2020). 10.1016/j.neuron.2019.10.030 PMC6975166 31813653

[6] Dudman J. T., Krakauer J. W., The basal ganglia: From motor commands to the control of vigor. Curr. Opin. Neurobiol. 37, 158–166 (2016). 27012960 10.1016/j.conb.2016.02.005

[7] Klaus A., Alves da Silva J., Costa R. M., What, if, and when to move: Basal ganglia circuits and self-paced action initiation. Annu. Rev. Neurosci. 42, 459–483 (2019). 31018098 10.1146/annurev-neuro-072116-031033

[8] Hikosaka O., Takikawa Y., Kawagoe R., Role of the basal ganglia in the control of purposive saccadic eye movements. Physiol. Rev. 80, 953–978 (2000). 10893428 10.1152/physrev.2000.80.3.953

[9] Jin X., Tecuapetla F., Costa R. M., Basal ganglia subcircuits distinctively encode the parsing and concatenation of action sequences. Nat. Neurosci. 17, 423–430 (2014). 24464039 10.1038/nn.3632 PMC3955116

[10] Howe M. W., Dombeck D. A., Rapid signalling in distinct dopaminergic axons during locomotion and reward. Nature 535, 505–510 (2016). 27398617 10.1038/nature18942 PMC4970879

[11] da Silva J. A., Tecuapetla F., Paixão V., Costa R. M., Dopamine neuron activity before action initiation gates and invigorates future movements. Nature 554, 244–248 (2018). 29420469 10.1038/nature25457

[12] Howe M.., Coordination of rapid cholinergic and dopaminergic signaling in striatum during spontaneous movement. eLife 8, e44903 (2019). 30920369 10.7554/eLife.44903 PMC6457892

[13] Xiao C.., Cholinergic mesopontine signals govern locomotion and reward through dissociable midbrain pathways. Neuron 90, 333–347 (2016). 27100197 10.1016/j.neuron.2016.03.028 PMC4840478

[14] Murakami M., Shteingart H., Loewenstein Y., Mainen Z. F., Distinct sources of deterministic and stochastic components of action timing decisions in rodent frontal cortex. Neuron 94, 908–919.e7 (2017). 28521140 10.1016/j.neuron.2017.04.040

[15] Hikosaka O., The habenula: From stress evasion to value-based decision-making. Nat. Rev. Neurosci. 11, 503–513 (2010). 20559337 10.1038/nrn2866 PMC3447364

[16] Mena-Segovia J., Bolam J. P., Rethinking the pedunculopontine nucleus: From cellular organization to function. Neuron 94, 7–18 (2017). 28384477 10.1016/j.neuron.2017.02.027

[17] O’Reilly J. X., Woolrich M. W., Behrens T. E. J., Smith S. M., Johansen-Berg H., Tools of the trade: Psychophysiological interactions and functional connectivity. Soc. Cogn. Affect. Neurosci. 7, 604–609 (2012). 22569188 10.1093/scan/nss055 PMC3375893

[18] Kim H. F., Hikosaka O., Parallel basal ganglia circuits for voluntary and automatic behaviour to reach rewards. Brain 138, 1776–1800 (2015). 25981958 10.1093/brain/awv134 PMC4492412

[19] Ghashghaei H. T., Barbas H., Neural interaction between the basal forebrain and functionally distinct prefrontal cortices in the rhesus monkey. Neuroscience 103, 593–614 (2001). 11274781 10.1016/s0306-4522(00)00585-6

[20] Monosov I. E., Anterior cingulate is a source of valence-specific information about value and uncertainty. Nat. Commun. 8, 134 (2017). 28747623 10.1038/s41467-017-00072-y PMC5529456

[21] Friston K., Causal modelling and brain connectivity in functional magnetic resonance imaging. PLoS Biol. 7, e33 (2009). 19226186 10.1371/journal.pbio.1000033 PMC2642881

[22] Heilbronner S. R., Hayden B. Y., Dorsal anterior cingulate cortex: A bottom-up view. Annu. Rev. Neurosci. 39, 149–170 (2016). 27090954 10.1146/annurev-neuro-070815-013952 PMC5512175

[23] Lara A. H., Elsayed G. F., Zimnik A. J., Cunningham J. P., Churchland M. M., Conservation of preparatory neural events in monkey motor cortex regardless of how movement is initiated. eLife 7, e31826 (2018). 30132759 10.7554/eLife.31826 PMC6112854

[24] Thaler D., Chen Y. C., Nixon P. D., Stern C. E., Passingham R. E., The functions of the medial premotor cortex. I. Simple learned movements. Exp. Brain Res. 102, 445–460 (1995). 7737391 10.1007/BF00230649

[25] Ledbetter N. M., Chen C. D., Monosov I. E., Multiple mechanisms for processing reward uncertainty in the primate basal forebrain. J. Neurosci. 36, 7852–7864 (2016). 27466331 10.1523/JNEUROSCI.1123-16.2016 PMC6601885

[26] Monosov I. E., Leopold D. A., Hikosaka O., Neurons in the primate medial basal forebrain signal combined information about reward uncertainty, value, and punishment anticipation. J. Neurosci. 35, 7443–7459 (2015). 25972172 10.1523/JNEUROSCI.0051-15.2015 PMC4429151

[27] Monosov I. E., Hikosaka O., Selective and graded coding of reward uncertainty by neurons in the primate anterodorsal septal region. Nat. Neurosci. 16, 756–762 (2013). 23666181 10.1038/nn.3398 PMC4160807

[28] Zhang K., Chen C. D., Monosov I. E., Novelty, salience, and surprise timing are signaled by neurons in the basal forebrain. Curr. Biol. 29, 134–142.e3 (2019). 30581022 10.1016/j.cub.2018.11.012 PMC6901356

[29] Bromberg-Martin E. S., Matsumoto M., Hikosaka O., Distinct tonic and phasic anticipatory activity in lateral habenula and dopamine neurons. Neuron 67, 144–155 (2010). 20624598 10.1016/j.neuron.2010.06.016 PMC2905384

[30] Hong S., Hikosaka O., Pedunculopontine tegmental nucleus neurons provide reward, sensorimotor, and alerting signals to midbrain dopamine neurons. Neuroscience 282, 139–155 (2014). 25058502 10.1016/j.neuroscience.2014.07.002 PMC4302061

[31] Baker P. M., Oh S. E., Kidder K. S., Mizumori S. J. Y., Ongoing behavioral state information signaled in the lateral habenula guides choice flexibility in freely moving rats. Front. Behav. Neurosci. 9, 295 (2015). 26582981 10.3389/fnbeh.2015.00295 PMC4631824

[32] Mizumori S. J. Y., Baker P. M., The lateral habenula and adaptive behaviors. Trends Neurosci. 40, 481–493 (2017). 28688871 10.1016/j.tins.2017.06.001 PMC11568516

[33] Syed A., Baker P. M., Ragozzino M. E., Pedunculopontine tegmental nucleus lesions impair probabilistic reversal learning by reducing sensitivity to positive reward feedback. Neurobiol. Learn. Mem. 131, 1–8 (2016). 26976089 10.1016/j.nlm.2016.03.010 PMC4862904

[34] Grabli D.., Gait disorders in parkinsonian monkeys with pedunculopontine nucleus lesions: A tale of two systems. J. Neurosci. 33, 11986–11993 (2013). 23864685 10.1523/JNEUROSCI.1568-13.2013 PMC6794061

[35] Kojima J.., Excitotoxic lesions of the pedunculopontine tegmental nucleus produce contralateral hemiparkinsonism in the monkey. Neurosci. Lett. 226, 111–114 (1997). 9159502 10.1016/s0304-3940(97)00254-1

[36] Lecourtier L., Kelly P. H., Bilateral lesions of the habenula induce attentional disturbances in rats. Neuropsychopharmacology 30, 484–496 (2005). 15562296 10.1038/sj.npp.1300595

[37] Lee E. H., Huang S. L., Role of lateral habenula in the regulation of exploratory behavior and its relationship to stress in rats. Behav. Brain Res. 30, 265–271 (1988). 3178997 10.1016/0166-4328(88)90169-6

[38] Berke J. D., What does dopamine mean? Nat. Neurosci. 21, 787–793 (2018). 29760524 10.1038/s41593-018-0152-y PMC6358212

[39] Dautan D.., A major external source of cholinergic innervation of the striatum and nucleus accumbens originates in the brainstem. J. Neurosci. 34, 4509–4518 (2014). 24671996 10.1523/JNEUROSCI.5071-13.2014 PMC3965779

[40] Gielow M. R., Zaborszky L., The input-output relationship of the cholinergic basal forebrain. Cell Rep. 18, 1817–1830 (2017). 28199851 10.1016/j.celrep.2017.01.060 PMC5725195

[41] Mesulam M.-M., Mufson E. J., Levey A. I., Wainer B. H., Cholinergic innervation of cortex by the basal forebrain: Cytochemistry and cortical connections of the septal area, diagonal band nuclei, nucleus basalis (substantia innominata), and hypothalamus in the rhesus monkey. J. Comp. Neurol. 214, 170–197 (1983). 6841683 10.1002/cne.902140206

[42] Pahapill P. A., Lozano A. M., The pedunculopontine nucleus and Parkinson’s disease. Brain 123, 1767–1783 (2000). 10960043 10.1093/brain/123.9.1767

[43] Mai J. K., Majtanik M., George P. A. N. D., Atlas of the Human Brain, (Academic Press, ed. 4, 2015).

[44] Holm S., A simple sequentially rejective multiple test procedure. Scand. J. Stat. 6, 65–70 (1979).

[45] Khalighinejad N., Priestley L., Jbabdi S., Rushworth M., Human decisions about when to act originate within a basal forebrain-nigral circuit, v4. Dryad. Available at 10.5061/dryad.prr4xgxhv. Deposited 23 April 2020. PMC7260969 32385157

