# Supplementary material for: Human decisions about when to act originate within a basal forebrain–nigral circuit
Source: Proc Natl Acad Sci U S A. 2020 May 8;117(21):11799–810. doi: 10.1073/pnas.1921211117 (PMC7260969; doi:10.1073/pnas.1921211117)
Supplement: Supplementary File [file pnas.1921211117.sapp.pdf]

Supporting Information

Experimental task. Participants were instructed to look at a screen and to make choices to maximise their reward. At the beginning of each trial a vertical rectangle appeared on the centre of the screen which we refer to as the 'water tank' (Fig.1A). The 'water' (the blue filling of the rectangle) level started dropping as soon as the trial started. As the water level was dropping 'bubbles' (transparent circles) emerged from the water. Each bubble remained on the screen for 2 s before popping and a new bubble emerging. It took 20 s for the whole 'tank' to drain (total number of bubbles in a tank = 9) (Fig.1B). Participants were told that bubbles might contain reward. They could gain monetary reward by choosing a bubble, at a time of their choice, by pressing the response key. The colour and the size of bubbles represented potential reward magnitude and reward probability, respectively. The colour of the bubbles changed from trial-to-trial but remained constant within a trial (within each water tank) and had three levels: the bubbles could be golden (20 p), silver (10 p), or bronze (5 p). The size of the bubbles changed within each trial with a constant rate: the bubbles got bigger and bigger or smaller and smaller, as the water level was dropping. The rate of change (slope) of size of the bubbles (reward probability) changed from trial-to-trial: the reward probability of the first bubble was fixed at 50%, gradually changing until the last bubble at 0, 20, 40, 60, 80, or 100%; meaning that in half of the trials the reward probability got higher and higher (bubbles got bigger and bigger), while in the other half reward probability got lower and lower (bubbles got smaller and smaller), the longer participants waited. Additionally, from trial-to-trial different levels of white Gaussian noise was added to the linear change in reward probability. The signal-to-noise ratio (SNR) had six levels: 15, 23, 34, 51 and 76 dB. Hence, while in some trials it was very easy for participants to track the rate of change in size of the bubbles (reward probability), it was much harder in other trials. Together these factors comprised the "present contextual factors" (Fig.1C). Importantly, they were varied independently of one another, and in a pseudo-randomised order. In addition to the present context, we also investigated whether the outcomes and

action times of recent past trials influenced participants' action time on the current trial (Fig.1D).

Participants were instructed to track the bubbles and to make a response by pressing on a response button at a time of their own choice. Once they responded the stimulus disappeared and a fixation cross appeared on the screen for the interval between response and outcome presentation. The duration of this interval was randomly drawn from a Gamma distribution (min 4 s, max 10 s, mean 5.5 s). During the outcome phase (2 s), if rewarded, a gold, silver, or bronze coin was shown on the screen, representing 20, 10, or 5 p, respectively. If not rewarded, or in rare occasions that participants did not make any response, a dark coin appeared on the screen. In addition to the reward on the current trial, the total reward earned, and the total time left in the experiment was also displayed on the screen at every outcome phase. After the outcome phase the fixation cross reappeared on the screen for the inter-trial-interval (ITI). The duration of the ITI was randomly drawn from a Gaussian distribution (mean = 4.5 s, std = 0.25 s) (Fig.1A).

Before the main task, participants were given written instructions and were trained on the task for 10 min. Once they were comfortable with the task the main experiment started inside the MRI scanner. The duration of each scanning session was set at 45 min. The task finished after 45 min, regardless of the number of trials performed. The experiment was written in Matlab (Mathworks, Natick, USA), using the Psychophysics Toolbox extension (1).

Behavioural analysis. Time to act (*actTime*) was defined as the natural logarithm of the time passed in seconds from beginning of the trial to the moment that participants made their response. We used a linear mixed-effect model (LMEM) to predict *actTime* from present and past contextual factors. The maximum likelihood method was used for model estimation. We examined the impact of both present and past contextual factors on *actTime*. Present contextual factors consisted of potential reward magnitude, change in reward

probability and noise on the current trial. Past contextual factors consisted of actual reward outcome and *actTime* on the past trial. All predictor variables were normalised.

$$actTime_t = \beta_0 + \beta_1 reward_t + \beta_2 probChange_t + \beta_3 noise_t + \beta_4(reward_t * probChange_t) + \beta_5(noise_t * probChange_t) + \beta_6 rewardOutcome_{t-1} + \beta_7 actTime_{t-1} + \beta_8 totalTime_t + \mu_0 + \mu_1 reward_t + \mu_2 probChange_t + \mu_3 noise_t + \mu_4(reward_t * probChange_t) + \mu_5(noise_t * probChange_t) + \mu_6 rewardOutcome_{t-1} + \mu_7 actTime_{t-1} + e,$$

where β_{0-8} are the fixed effects, μ_0 is by-subject random intercept, and μ_{1-7} are by-subject random slopes. Total time passed from beginning of the testing session (*totalTime*) was added to the model as a covariate of no interest. The modelling was performed with the 'lme4' package in R (2).

Cox regression model. To estimate the deterministic component of *actTime* we used a specific class of survival models called the Cox proportional hazard model (3, 4). The model predicts time-to-event (*actTime*) on the current trial from present and past contextual factors. Specifically, the predictors (covariates) included reward magnitude, change in reward probability, and ITI of the current trial, and the actual reward and *actTime* on the past 10 trials. The model is described as:

$$\lambda(t) = \lambda_0(t).\exp(\beta\mathbf{x}),$$

where $\lambda(t)$represents a hazard function (hazard rate of responding), $\lambda_0(t)$ represents a baseline hazard function, that is a hazard function when all the covariates are 0, β is a row vector with 23 elements (3 present contextual factors + 10 past rewards + 10 past *actTimes*) representing Cox coefficients for each covariate and **x** is a 23 element column vector representing covariates, present contextual factors and contextual factors of the past 10 trials. The coefficients were estimated for each testing session by using the 'coxphfit' function in MATLAB.

A detailed method for obtaining Cox coefficients has been previously described (4). The estimated Cox coefficients ($\hat{\beta}$) from the predictors on the current trial and the immediately preceding trial were used to obtain the expected *actTime* by the following method: First, the cumulative hazard function, $\widehat{\Lambda}_x(t)$, of each trial was estimated given the baseline cumulative hazard function, $\widehat{\Lambda}_0(t)$, and the covariates:

$$\widehat{\Lambda}_x(t) = \widehat{\Lambda}_0(t).\exp(\hat{\beta}\mathbf{x}),$$

The cumulative hazard function of each trial was then used to estimate the survival function of each trial, $S(t)$:

$$\hat{S}_x(t) = \exp\ (-\widehat{\Lambda}_x(t)),$$

The deterministic *actTime* is estimated by:

$$[actTime] = \int_0^{\infty} \hat{S}_x\ (t),$$

Finally, to measure the proportion of variance explained by the Cox regression model, we used Schemper's *V* (5), which is defined as:

$$V = \frac{(\widehat{D} - \widehat{D}_x)}{\widehat{D}},$$

Where $\widehat{D}$ is the distance between survival functions of individual trials $S_i(t)$ and a survival function estimated from all the trials without taking into account covariates $\hat{S}(t)$, by using Kaplan–Meier estimator. $\widehat{D}_x$ is calculated in the same way as $\widehat{D}$, but is the distance between survival functions of individual trials $S_i(t)$, and an estimated conditional survival function given covariates **x**, $\hat{S}_x(t)$. The equations to calculate $\widehat{D}$ are previously described in detail (4).

Imaging data acquisition. Structural and functional MRI was collected using a Siemens 7 T MRI scanner. High-resolution functional data was acquired using a multiband gradient-echo

T2* echo planar imaging (EPI) sequence with a 1.2 x 1.2 x 1.2 mm resolution; multiband acceleration factor 3; repetition time (TR) 1.5 s; echo time (TE) 21.8 ms; flip angle 60°; and a GRAPA acceleration factor 3. To compensate for the high spatial and temporal resolution we used a limited field of view (FOV) oriented at approximately 75° with respect to the AC-PC line (coronal orientation with foot-to-head phase encoding direction; 72 slices; coverage 86.4 mm). The posterior edge of the FOV was positioned at the back of the splenium of corpus callosum. This specific position covered all our regions of interest including motor cortex, premotor cortex, supplementary motor area, cingulate cortex, basal ganglia, basal forebrain, midbrain and brainstem. Additionally, a single-measurement, whole-brain, functional image was acquired prior to the main functional image (with similar orientation). This was later used for registration of the main functional image with limited FOV to the whole brain. Structural data was acquired with a T1-weighted MP-RAGE sequence with a 1 x 1 x 1 mm resolution; GRAPPA acceleration factor of 2; TR 2200 ms; TE 2.82 ms; and inversion time (TI) 1050 ms. To correct for field inhomogeneities a separate Fieldmap sequence was acquired with a 2 x 2 x 2 mm resolution; TR 620 ms; TE1 4.08 ms; TE2 5.10 ms. Finally, to regress out the effect of physiological noise in functional data, cardiac and respiratory frequencies were collected by pulse oximetry and respiratory bellows.

fMRI data processing. Preprocessing was performed using tools from FMRIB Software Library (FSL) (6). Functional images were first normalised, spatially smoothed (Gaussian kernel with 2.5mm full-width half-maximum), and temporally high-pass filtered (3 dB cut-off of 100 s). The effect of participants' head motion during the scanning was removed by MCFLIRT (7). The Brain Extraction Tool (8) was used on functional and structural images to separate brain from non-brain matter. The registration of functional images into Montreal Neurological Institute (MNI)-space was performed in three stages: (1) Limited FOV EPI to whole-brain EPI using FMRIB's Linear Image Registration Tool (9) with 3 degrees of freedom (translation only). (2) Whole-brain EPI to individual structural image using Boundary-Based Registration (BBR) (10) by incorporating Fieldmap correction. (3) Individual

structural image to Standard image by using FMRIB's Non-linear Image Registration Tool (FNIRT).

Whole-brain fMRI data analyses. Whole-brain statistical analyses was performed at two-levels as implemented in FSL FEAT (11). At the first level, we used a univariate general linear model (GLM) framework for each participant to compute the parameter estimates. The contrast of parameter estimates and variance estimates from each scanning session were then combined in a second-level mixed-effects analysis (FLAME 1+2) (12), treating scanning sessions as random effect. The results were cluster-corrected with the voxel inclusion threshold of Z = 3.1 and cluster significance threshold of P = 0.05. The data were pre-whitened before analysis to account for temporal autocorrelations (11).

For the first level analysis we looked for brain areas in which activity reflected parametric variation in the empirically observed *actTime*. Importantly, the GLM only included *actTime* and not all the present and past contextual factors that influenced *actTime*.

$$GLM1: BOLD = \beta_0 + \beta_1\, stim + \beta_2\, totaltime + \beta_3\, actTimeLong + \beta_4\, actTimeShort + \beta_5\, mainAct + \beta_6\, mainOut + \beta_7\, reward,$$

where $BOLD$ is a t x 1 (t time samples) column vector containing the times series data for a given voxel. *stim* is an unmodulated regressor representing the main effect of stimulus presentation (all event amplitudes set to one). *totaltime* is a parametric regressor representing the time passed since the beginning of the scanning session. *actTimeLong* and *actTimeShort* are parametric regressors representing *actTime* on trials where rate of change in reward probability was positive (long *actTime* was the correct strategy) and negative (short *actTime* was the correct strategy), respectively. *mainAct* is an unmodulated regressor representing the main effect of responding. *mainOut* is an unmodulated regressor representing the main effect of outcome. *reward* is a parametric regressor with four levels (large, medium, low and no-reward) representing the reward outcome on the current trial. All

regressors were modelled as a boxcar function with constant duration of 1 s convolved with a double-gamma hemodynamic response function (HRF). Regressors 1-2 were time-locked to the onset of the trial. Regressor 3-5 started 1 s before participants made a response by pressing the response key and continued for 1 s. Regressors 6-7 were time-locked to the onset of the outcome phase. On the rare occasions where participants made no response, those trials were left out of the analysis (0.35%±0.88% of the trials, across all participants).

To further reduce variance and noise in the BOLD signal, we also added task-unrelated confounds which included: (1) head motion parameters as estimated by MCFLIRT in the pre-processing stage; (2) voxelwise regressors created by physiological noise modelling (PNM) (13) to model the effects of physiological noise (cardiac and respiratory); (3) regressors to completely remove timepoints with large motions that could not be fixed with linear methods.

Structural equation modelling. PPI analysis is applicable to a maximum of two ROIs at a time (14). To investigate how regions are connected at a wider circuit level, we conducted structural equation modelling (SEM) to probe for covariance between regions in the time-course of BOLD response. SEM is a well-established method for analysing functional and effective connectivity (14, 15). It assesses interrelationships among several continuous variables based on their covariance with one another. Importantly it defines the strength of connections between brain areas in question, rather than activity in individual variables. In practice, it is reminiscent of linear regression save that more complex patterns of relationships can be tested. As implemented here, it has the following formal definition:

$$\eta_t = \eta_t \cdot \beta + \zeta_t$$

where η_t is a matrix comprising the filtered time-series of BOLD response in the ROIs, β is a vector of path coefficients describing the relationship(s) between ROIs, and ζ_t is measurement error. Importantly, SEM estimates only contemporaneous relationships

between regions. It is customary in the social sciences to incorporate temporally lagged effects where possible, and the same strategy is suggested for BOLD time series data – the idea being that inter-temporal connections make for stronger claims about the causal direction of relationships between regions (16, 17). However, several intrinsic properties of BOLD response prevent straightforward interpretation of lagged relationships (18). They were therefore omitted from consideration. All structural equation modelling was conducted in Latent Variable Analysis (lavaan) package v.0.6–4 using Maximum Likelihood estimation. We evaluated model performance on the basis of three fit indices specific to structural equation models which reflect the absolute goodness-of-fit to the data; the goodness-of-fit index (GFI, where ≥ 0.95 indicates a good fit), the standardised root mean square residual (SRMR, where < 0.08 indicates a good fit) and the root mean square error of approximation (RMSEA, where < 0.07 indicates a good fit) (For formal descriptions of these indices and their interpretation see (19, 20)).

SI References

1. M. Kleiner, *et al.*, What's new in psychtoolbox-3. *Perception* **36**, 1–16 (2007).
2. D. Bates, *et al.*, *lme4: Linear Mixed-Effects Models using "Eigen" and S4* (2018) (December 13, 2018).
3. N. Khalighinejad, *et al.*, A Basal Forebrain-Cingulate Circuit In Macaques Decides It Is Time To Act. *Neuron* **0** (2019).
4. M. Murakami, H. Shteingart, Y. Loewenstein, Z. F. Mainen, Distinct Sources of Deterministic and Stochastic Components of Action Timing Decisions in Rodent Frontal Cortex. *Neuron* **94**, 908-919.e7 (2017).
5. M. Schemper, R. Henderson, Predictive accuracy and explained variation in Cox regression. *Biometrics* **56**, 249–255 (2000).
6. M. Jenkinson, C. F. Beckmann, T. E. J. Behrens, M. W. Woolrich, S. M. Smith, FSL. *NeuroImage* **62**, 782–790 (2012).
7. M. Jenkinson, P. Bannister, M. Brady, S. Smith, Improved optimization for the robust and accurate linear registration and motion correction of brain images. *NeuroImage* **17**, 825–841 (2002).
8. S. M. Smith, Fast robust automated brain extraction. *Hum. Brain Mapp.* **17**, 143–155 (2002).

9. M. Jenkinson, S. Smith, A global optimisation method for robust affine registration of brain images. *Med. Image Anal.* **5**, 143–156 (2001).

10. D. N. Greve, B. Fischl, Accurate and robust brain image alignment using boundary-based registration. *NeuroImage* **48**, 63–72 (2009).

11. M. W. Woolrich, B. D. Ripley, M. Brady, S. M. Smith, Temporal autocorrelation in univariate linear modeling of FMRI data. *NeuroImage* **14**, 1370–1386 (2001).

12. C. F. Beckmann, M. Jenkinson, S. M. Smith, General multilevel linear modeling for group analysis in FMRI. *NeuroImage* **20**, 1052–1063 (2003).

13. J. C. W. Brooks, *et al.*, Physiological noise modelling for spinal functional magnetic resonance imaging studies. *NeuroImage* **39**, 680–692 (2008).

14. K. J. Friston, Functional and Effective Connectivity: A Review. *Brain Connect.* **1**, 13–36 (2011).

15. A. R. McLntosh, F. Gonzalez-Lima, Structural equation modeling and its application to network analysis in functional brain imaging. *Hum. Brain Mapp.* (1994) https:/doi.org/10.1002/hbm.460020104 (August 4, 2019).

16. K. M. Gates, P. C. M. Molenaar, Group search algorithm recovers effective connectivity maps for individuals in homogeneous and heterogeneous samples. *NeuroImage* **63**, 310–319 (2012).

17. B. Zinszer, Effective Connectivity Modeling with the euSEM and GIMME. 11 (2013).

18. K. Friston, Causal Modelling and Brain Connectivity in Functional Magnetic Resonance Imaging. *PLoS Biol.* **7** (2009).

19. K. A. Bollen, J. S. Long, *Testing Structural Equation Models* (SAGE, 1993).

20. L. Hu, P. M. Bentler, Cutoff criteria for fit indexes in covariance structure analysis: Conventional criteria versus new alternatives. *Struct. Equ. Model. Multidiscip. J.* **6**, 1–55 (1999).

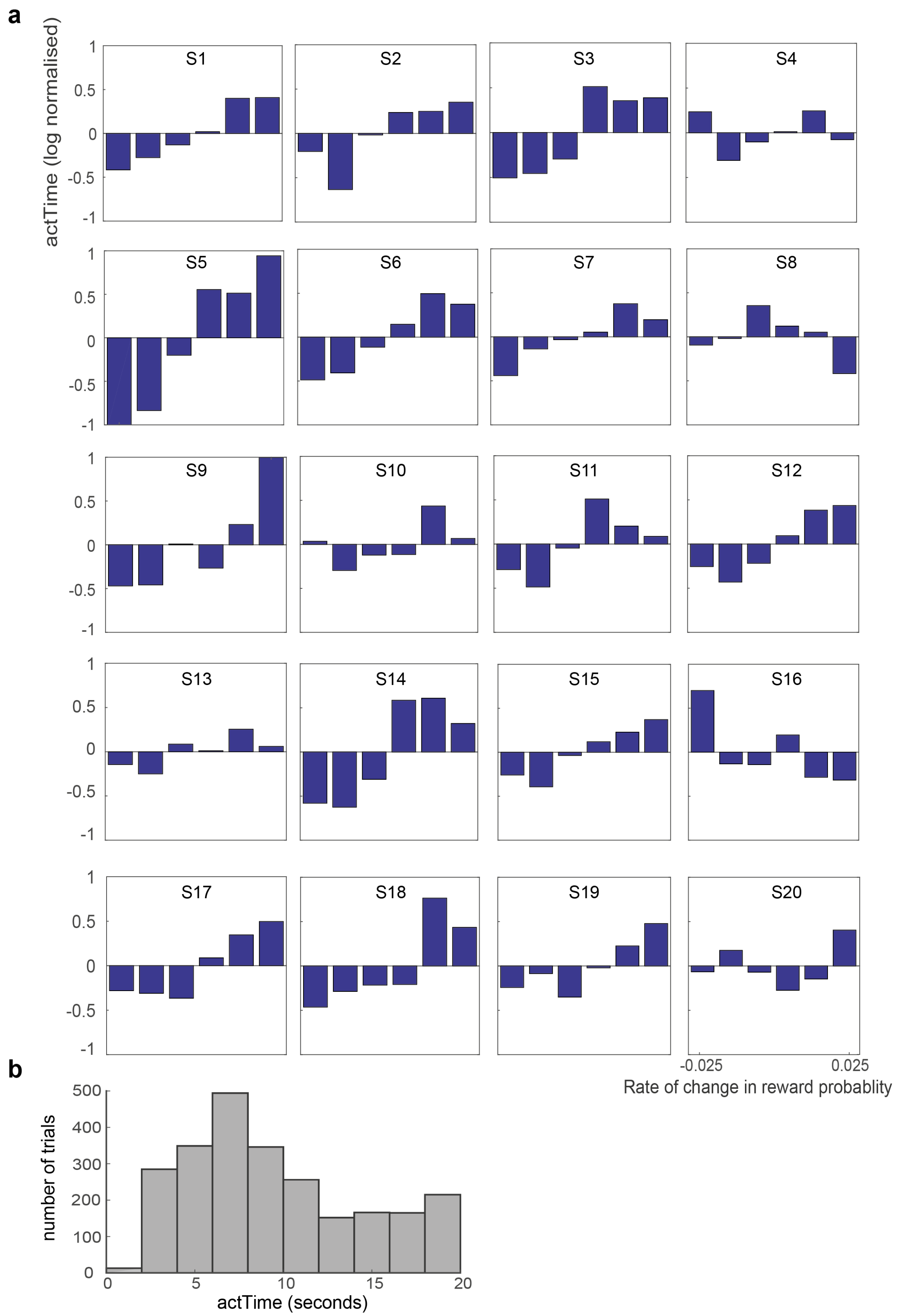

Figure S1. Related to Figure 2a. (a) Format as in Fig.2A but with data from each participant reported separately. (b) Distribution of action times in seconds across all trials.

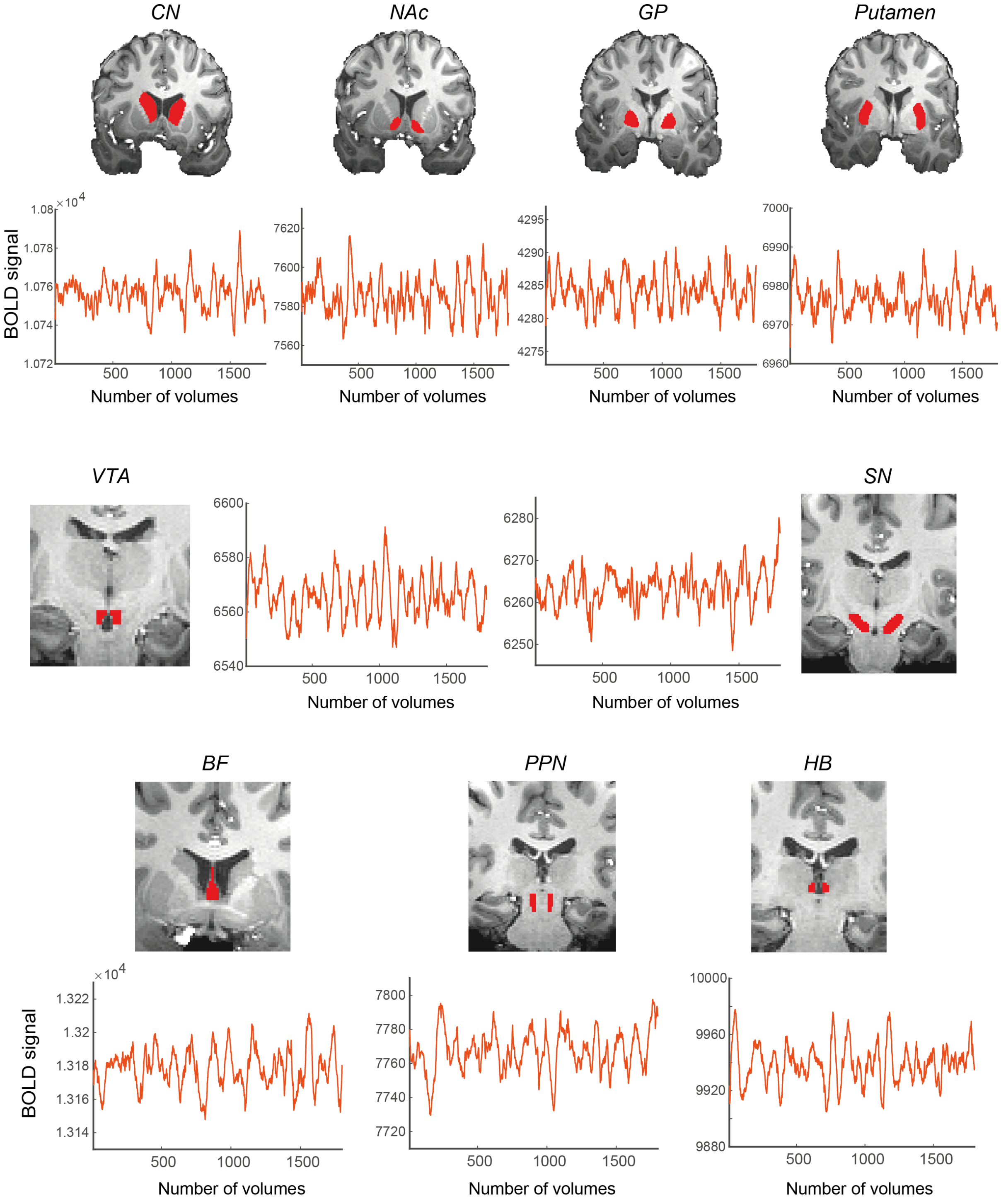

CN
NAc
GP
Putamen
BOLD signal
×10^4
1.08
1.078
1.076
1.074
1.072
7620
7600
7580
7560
4295
4290
4285
4280
4275
7000
6990
6980
6970
6960
500
1000
1500
Number of volumes
VTA
SN
6600
6580
6560
6540
6280
6270
6260
6250
BF
PPN
HB
×10^4
1.322
1.32
1.318
1.316
1.314
7800
7780
7760
7740
7720
10000
9960
9920
9880

Figure S2. Related to Figure 3. Anatomical masks were designed for each ROI in the MNI standard space and were transformed from the standard space to each participant's structural space by applying a standard-to-structural warp. To make sure that the masks still match the ROIs' boundaries after unwarping, they were manually edited within each participant's structural space using FSLeyes. Edited anatomical masks from an example subject (S12) are shown here. The masks are overlaid on structural image of the subject. The extracted BOLD signals from each mask is displayed next to its corresponding ROI. The lines show the BOLD signal extracted and averaged from each voxel within the ROI, for the whole duration of the scanning session.

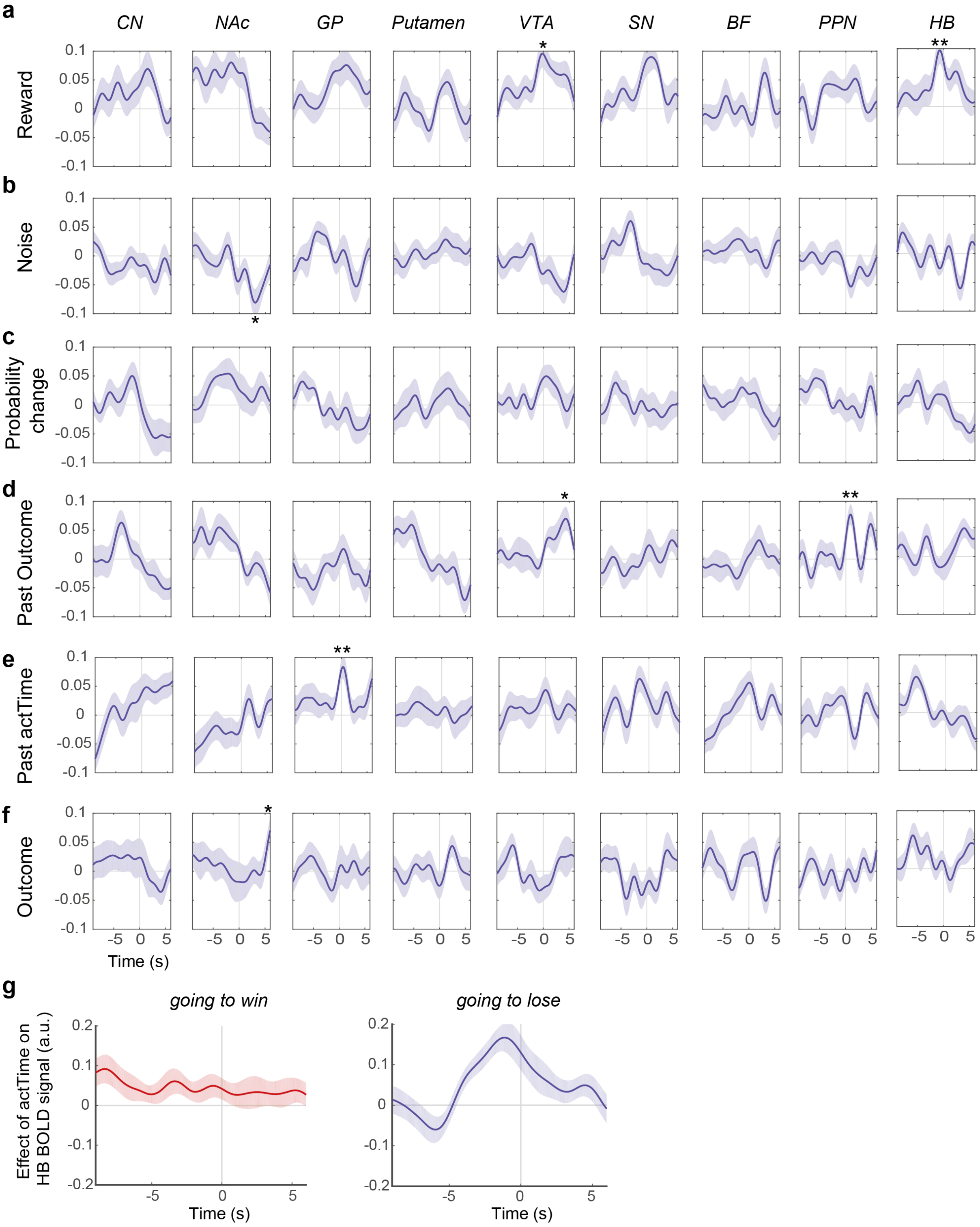
a
b
c
d
e
f
g
CN
NAc
GP
Putamen
VTA
SN
BF
PPN
HB
Reward
Noise
Probability change
Past Outcome
Past actTime
Outcome
Time (s)
going to win
going to lose
Effect of actTime on HB BOLD signal (a.u.)

Figure S3. Related to Figure 3. ROI time-course analysis of the CN (caudate nucleus), NAc (nucleus accumbens), GP (globus pallidus), VTA (ventral tegmental area), SN (substantia nigra), BF (basal forebrain), PPN (pedunculopontine nucleus), and HB (habenula) showing the relationship between BOLD activity and parametric variation in reward magnitude (a), noise level (b), rate of change in reward probability (c), past reward outcome (d), past *actTime* (e), and outcome on the current trial (f). We found individual effect of contextual factors on NAc (reward outcome and noise level on the current trial), GP (past *actTime*), VTA (expected reward magnitude on the current trial and reward outcome on the past trial), PPN (reward outcome on the past trial), and HB (expected reward magnitude). Previous studies have shown a negative relationship between expected reward and HB activity (15). It is thus surprising to find a positive relationship between HB BOLD activity and reward magnitude (a). We therefore performed further analyses to investigate the relationship between HB activity, reward prospect, and action time. Interestingly, we found that the positive relationship between HB BOLD signal and action time (Fig.3) is mainly driven by trials on which participants were expecting to lose (on trials where rate of change in reward probability was negative) (g). This finding corresponds with the previous literature that HB is more active when a loss or an aversive event is predicted. The lines and shadings show the mean and standard error of the β weights across the participants, respectively. Time zero is the response time. Significance testing on time-course data was performed by using a leave-one-out procedure on the group peak signal. One-sample t-tests with Holm-Bonferroni correction. * $P<0.05$, ** $P<0.01$, *** $P<0.001$.

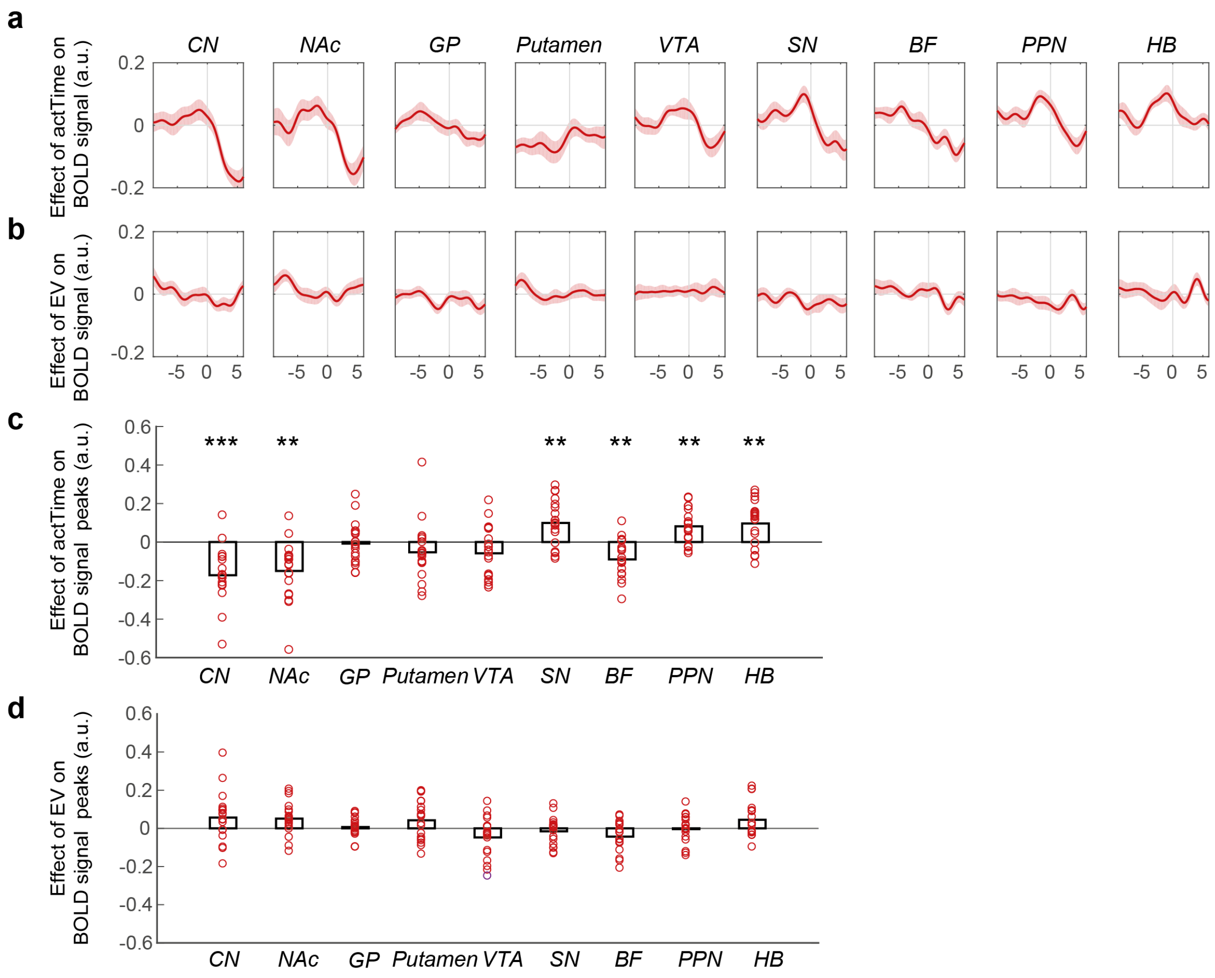

Figure S4. Related to Figure 3. The trial-by-trial variance in rate of change in reward probability decorrelates expected reward from action time. Nevertheless, to make sure that our results could not be merely explained by the expected value (EV) we measured EV for each trial by multiplying the reward magnitude (inferred from the colour of the bubbles) by reward probability (inferred from the size of the bubble at the time of response) and added it as a confound regressor to the main model (GLM2.1). The effect of *actTime* on BOLD activity (a,c) is similar to the original results (compare panel c with Fig.3B). However, we found no significant relationship between EV and BOLD activity (b,d). Format as in Figure 3.

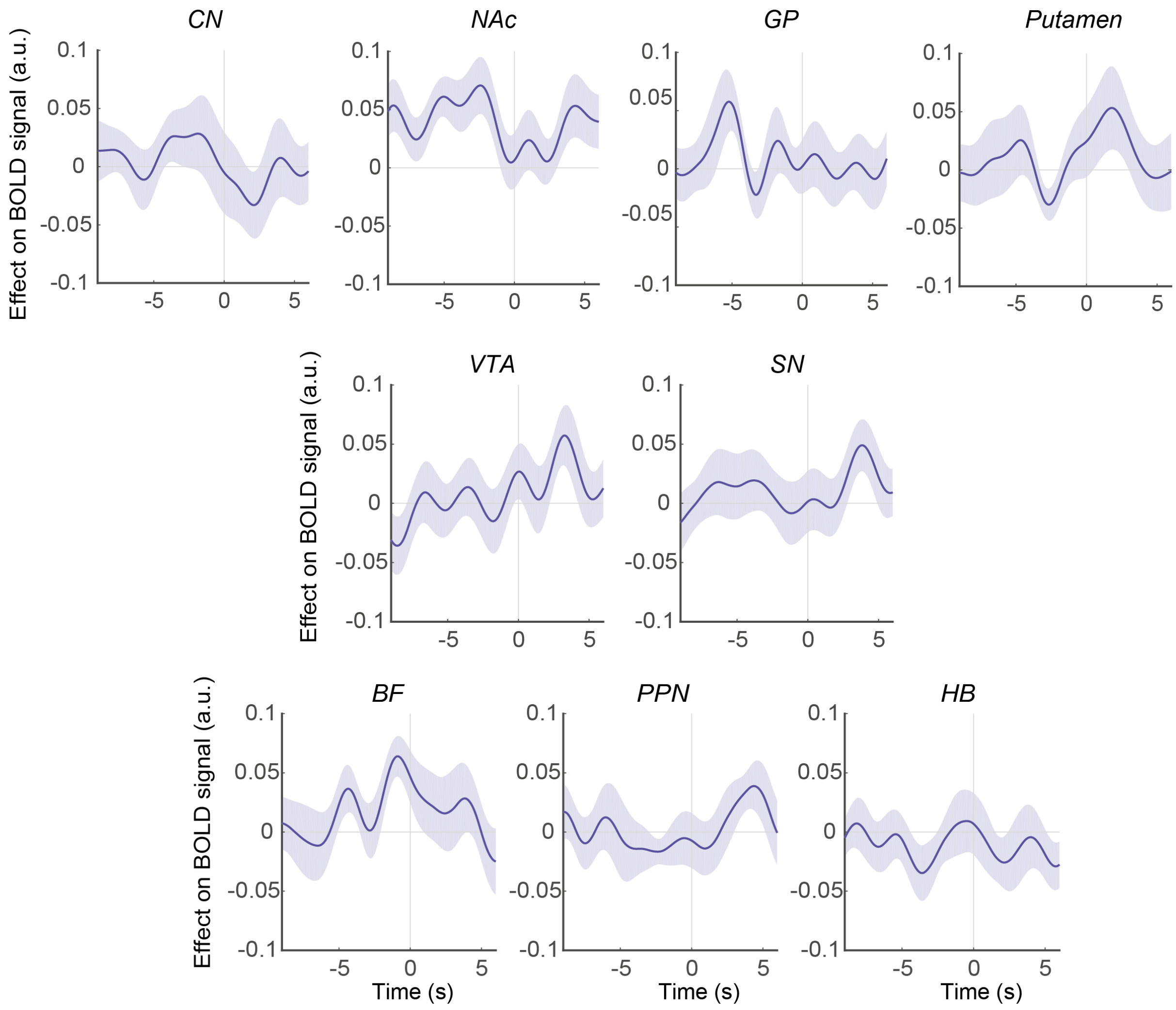

Figure S5. Related to Figure 5. ROI time-course analysis of the ROIs, showing the relationship between BOLD activity and deterministic *actTime* estimated from both present and past contextual factors. The lines and shadings show the mean and standard error of the β weights across the participants, respectively. Time zero is the response time.

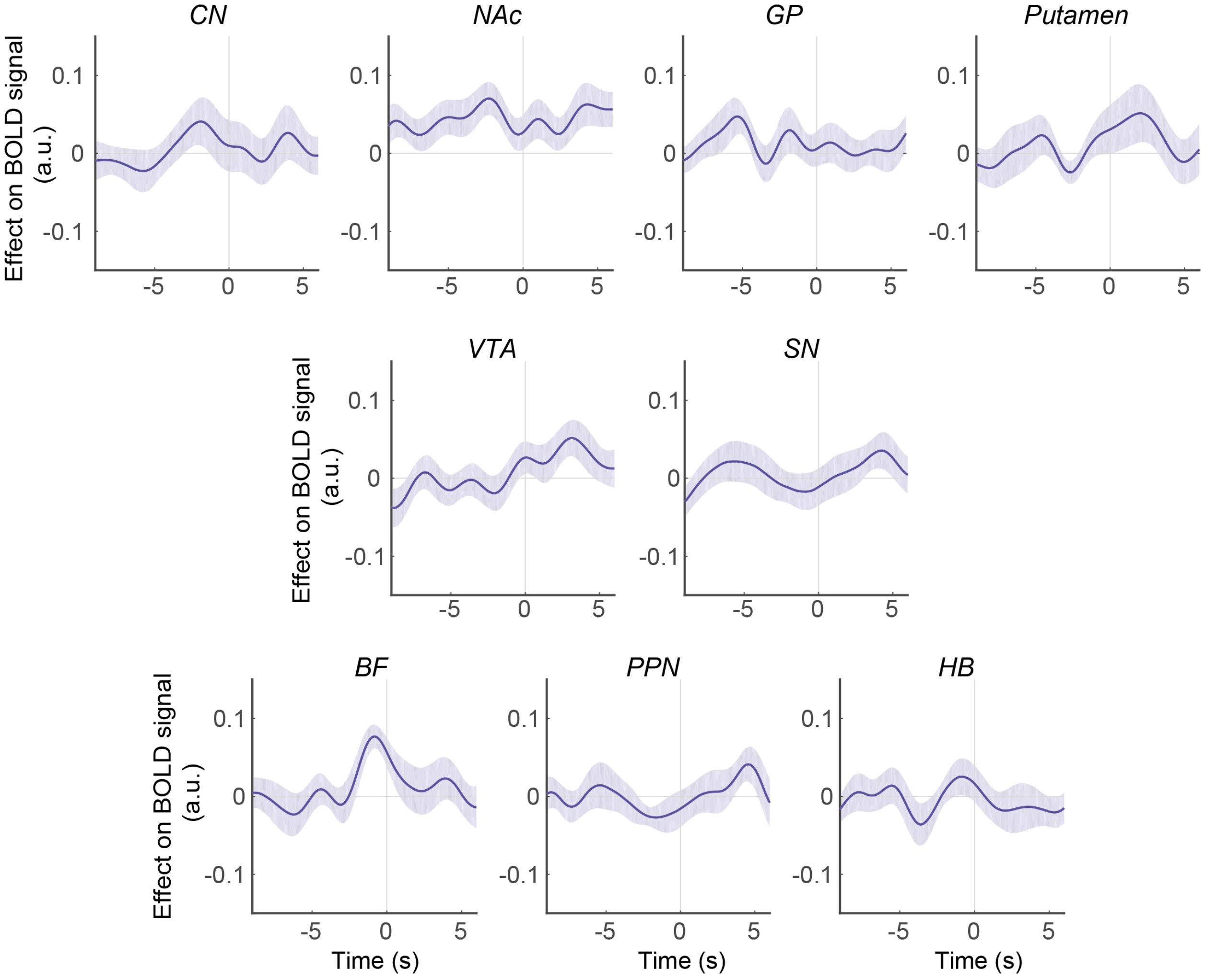

Figure S6. Related to Figure 5. ROI time-course analysis of the ROIs, showing the relationship between BOLD activity and deterministic *actTime* estimated from present contextual factors. Format as in Fig.S5.

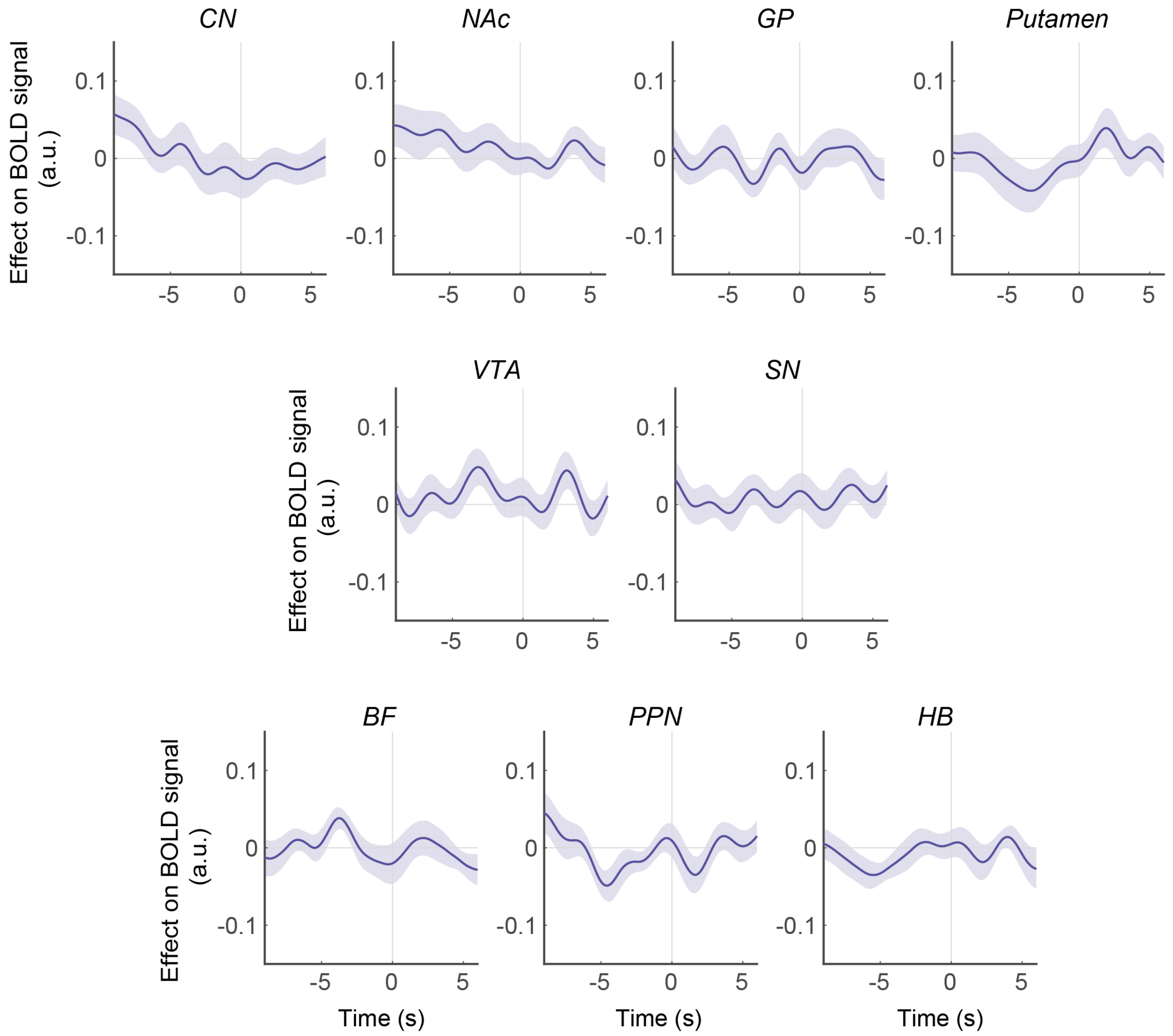

Figure S7. Related to Figure 5. ROI time-course analysis of the ROIs, showing the relationship between BOLD activity and deterministic *actTime* estimated from past contextual factors. Format as in Fig.S5.

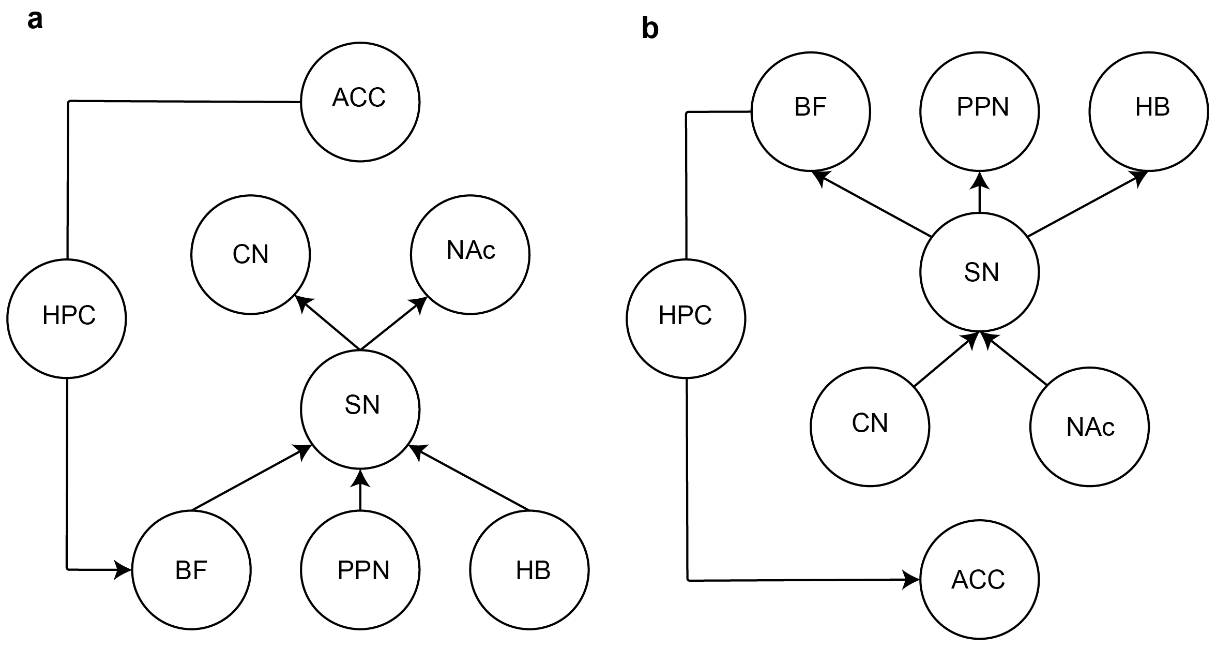

Figure S8. Related to Figure 7. The main direct projections from ACC to BF end in nucleus basalis. However, the BF mask that we used in this study mainly covers medial septal and diagonal nuclei. The hippocampus (HPC), however, provides a major input to the septal nuclei and septal nuclei projects back to the hippocampus. We therefore updated our model (Fig.7) by adding the BOLD activity from hippocampus to the pathway between ACC and BF (a). We found a significant path from ACC to hippocampus (β=-0.17, P<0.001) and from hippocampus to BF (β=0.21, P<0.001). We then compared the updated model with an alternative model with identical number of degrees of freedom but with the direction of paths reversed (b). The updated model (AIC=474288) performed better than the control model (AIC=583003). However, the updated model was no better than our original model (AIC=377600) at explaining the data.

Long actTime contrast						
**Cluster**	**No. of voxels**	**P value**	**Z-max**	**X (mm)**	**Y (mm)**	**Z (mm)**
Cingulate gyrus	197	9.48E-20	4.35	0	-4	58
Middle frontal gyrus (right)	135	4.9E-15	4.60	26	-4	50
Postcentral gyrus (right)	110	6.16E-13	4.28	60	-16	28
Fusiform gyrus (right)	84	1.42E-10	4.41	34	-52	-20
Precentral gyrus (right)	55	1.19E-07	3.98	44	-2	50
Precentral gyrus (right)	46	1.31E-06	4.15	52	4	38
Supramarginal gyrus (left)	41	5.13E-06	4.05	-64	-28	36
Cerebellum	36	2.12E-05	4.71	-14	-54	-46
Fusiform gyrus (left)	35	2.83E-05	4.01	-36	-56	-16
Lingual gyrus (right)	26	0.0005	3.76	22	-42	-12
Precentral gyrus (left)	26	0.0005	4.03	-26	-8	60
Short actTime contrast						
**Cluster**	**No. of voxels**	**P value**	**Z-max**	**X (mm)**	**Y (mm)**	**Z (mm)**
Putamen (right)	118	3.82E-14	4.42	14	8	-8
Caudate (left)	55	5.96E-08	4.06	-10	12	-2

Table S1. Related to Figure 4. Full list of clusters related to *long actTime* and *short actTime* contrasts. Here we focussed on the largest cluster (Cingulate). Harvard-Oxford Cortical and Subcortical Structural Atlas was used for labelling.

SEM path coefficients			
**Path**	*β*	*SE*	*P*
**ACC → BF**	-0.118	0.005	<.001
**BF → SN**	0.051	0.005	<.001
**PPN → SN**	0.388	0.005	<.001
**HB → SN**	0.102	0.005	<.001
**SN → CN**	0.043	0.005	<.001
**SN → NAc**	0.037	0.005	<.001

Table S2. Related to Figure 7. Estimates of the path coefficients of SEM. CN (caudate nucleus), NAc (nucleus accumbens), SN (substantia nigra), BF (basal forebrain), PPN (pedunculopontine nucleus), HB (habenula). The arrows show the direction of the influence.
